# Hippocampal–prefrontal coherence mediates working memory and selective attention at distinct frequency bands and provides a causal link between schizophrenia and its risk gene *GRIA1*

**DOI:** 10.1038/s41398-019-0471-0

**Published:** 2019-04-18

**Authors:** Alexei M. Bygrave, Thomas Jahans-Price, Amy R. Wolff, Rolf Sprengel, Dimitri M. Kullmann, David M. Bannerman, Dennis Kätzel

**Affiliations:** 1 Department of Experimental Psychology, University of Oxford, Oxford, UK; 2 UCL Queen Square Institute of Neurology, University College London, London, UK; 3 Max-Planck-Institute for Medical Research, Heidelberg, Germany; 4 Institute for Anatomy and Cell Biology, Heidelberg University, Heidelberg, Germany; 5 Institute of Applied Physiology, Ulm University, Ulm, Germany; 6 Present Address: Department of Neuroscience, Johns Hopkins University, Baltimore, MD USA

**Keywords:** Molecular neuroscience, Physiology, Diagnostic markers

## Abstract

Increased fronto-temporal theta coherence and failure of its stimulus-specific modulation have been reported in schizophrenia, but the psychological correlates and underlying neural mechanisms remain elusive. Mice lacking the putative schizophrenia risk gene *GRIA1* (*Gria1*^–/–^), which encodes GLUA1, show strongly impaired spatial working memory and elevated selective attention owing to a deficit in stimulus-specific short-term habituation. A failure of short-term habituation has been suggested to cause an aberrant assignment of salience and thereby psychosis in schizophrenia. We recorded hippocampal–prefrontal coherence while assessing spatial working memory and short-term habituation in these animals, wildtype (WT) controls, and *Gria1*^–/–^ mice in which GLUA1 expression was restored in hippocampal subfields CA2 and CA3. We found that beta (20–30 Hz) and low-gamma (30–48 Hz) frequency coherence could predict working memory performance, whereas—surprisingly—theta (6–12 Hz) coherence was unrelated to performance and largely unaffected by genotype in this task. In contrast, in novel environments, theta coherence specifically tracked exploration-related attention in WT mice, but was strongly elevated and unmodulated in *Gria1*-knockouts, thereby correlating with impaired short-term habituation. Strikingly, reintroduction of GLUA1 selectively into CA2/CA3 restored abnormal short-term habituation, theta coherence, and hippocampal and prefrontal theta oscillations. Although local oscillations and coherence in other frequency bands (beta, gamma), and theta-gamma cross-frequency coupling also showed dependence on GLUA1, none of them correlated with short-term habituation. Therefore, sustained elevation of hippocampal–prefrontal theta coherence may underlie a failure in regulating novelty-related selective attention leading to aberrant salience, and thereby represents a mechanistic link between *GRIA1* and schizophrenia.

## Introduction

It has been proposed that long-range communication between brain regions can be flexibly reconfigured by episodes of synchrony or coherence of neural activity in different brain areas^1^. This coherence between the local oscillations in interacting regions might enable the flexible routing of information between them^2–4^. Abnormalities in long-range coherence have been identified in patients with schizophrenia^5–9^ and may underlie both cognitive and positive symptoms^6,10^. For example, studies in patients with schizophrenia have found abnormally increased baseline theta frequency coherence between multiple brain areas, whereby elevated fronto-temporal theta coherence correlates with positive symptom scores^8,9,11^. Moreover, those patients show a failure to transiently modulate theta coherence in response to sensory stimuli^11^. However, the causes of abnormal coherence in schizophrenia remain elusive.

Impaired glutamatergic signaling and synaptic plasticity in the hippocampus are strongly linked to schizophrenia^12–14^. For example, *GRIA1*, the gene encoding the α-amino-3-hydroxy-5-methyl-4-isoxazolepropionic acid (AMPA) receptor subunit GLUA1, has been identified as a putative risk gene for schizophrenia in recent GWAS^15,16^. Furthermore, decreased mRNA and protein levels of *GRIA1* have also been found in post-mortem hippocampal tissue from schizophrenia patients^17,18^.

*Gria1*-knockout mice (*Gria1*^–/–^) are therefore a key experimental tool for investigating the role of GLUA1 dysfunction and impaired GLUA1-dependent synaptic plasticity in schizophrenia^13,19–21^. They show robust, yet selective, impairments in tasks that rely on hippocampus-dependent short-term memory^22–24^, whereas general spatial processing, long-term habituation, and long-term associative learning are left intact^24–26^. For example, *Gria1-*knockout mice lack spatial working memory, even after extensive training^24,27^. They also display chance level performance in the spatial novelty preference (SNP) Y-maze test^26^, and show pronounced and sustained novelty-induced locomotor hyperactivity^20,27–29^. The latter phenotypes are likely driven by their failure to reduce levels of attention paid to novel stimuli when they would normally become familiar, i.e., they display impaired short-term habituation^13,22^.

Here, we made simultaneous local field potential (LFP) recordings in the dorsal hippocampus (dHipp) and medial prefrontal cortex (PFC) of wildtype (WT) and *Gria1*^–/–^ mice in order to assess the importance of GLUA1 for hippocampal–prefrontal coherence. Given the convergence of multiple lines of evidence on a specific hippocampal role in the control of short-term habituation in rodents^30–32^ and patients with schizophrenia^33^, we additionally used a local viral rescue approach to restore GLUA1 expression in the hippocampus of *Gria1*^–/–^ mice. Thereby, we show that hippocampal GLUA1 expression plays a key role in regulating hippocampal–prefrontal theta coherence, which parallels its role in regulating selective attention through short-term habituation.

## Methods

### Animals and surgery

**Fig. 1:**
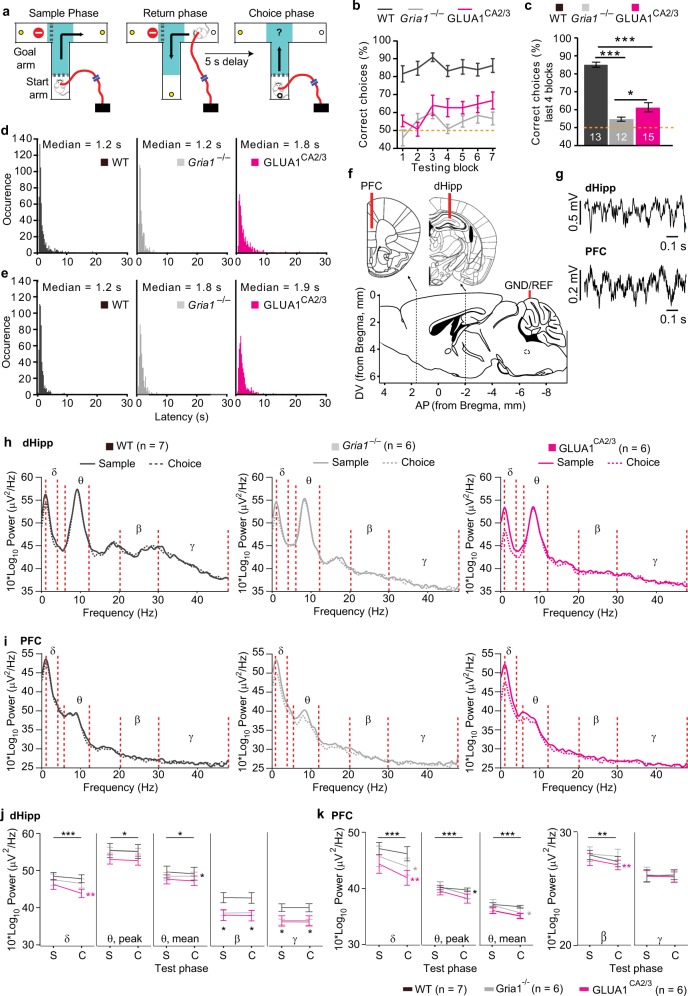
Behavioral performance and local oscillations in dHipp and PFC during T-maze spatial working memory. **a** Schematic of the phases of the T-maze, with the “decision zone” indicated by turquoise shading. **b** Spatial working memory (SWM) performance over the seven testing blocks in WT (black), *Gria1*^–/–^ (gray), and GLUA1^CA2/3^ (magenta) animals and **c** SWM performance averaged over the last four testing blocks. Stars indicate pairwise differences (Tukey post-hoc test, conducted after significant effect of group in ANOVA). **d**, **e** Histograms showing the latency during the sample **d** and choice **e** phase for all T-maze trials. **f** Schematic of electrode placement and **g** example traces of LFP recordings from the dorsal hippocampus (dHipp) and prefrontal cortex (PFC). **h**, **i** Group-average power spectra showing sample (solid lines) and choice phase (dashed lines) oscillatory power changes in the dorsal hippocampus **h** and prefrontal cortex **i**. Frequency bands for delta (1–4 Hz; *δ*), theta (6–12 Hz; *θ*), beta (20–30 Hz; *β*), and gamma (30–48 Hz; *γ*) are indicated between red dotted lines. **j**, **k** Quantification of mean delta (*δ*), peak theta (*θ*), mean theta (*θ*), mean beta (*β*) and mean gamma (*γ*) power in the dHipp **j** and PFC **k** in the sample (S) and choice (C) task phase, as indicated. Stars on top line in each subpanel indicate significant effect of test phase across all three groups (repeated-measures ANOVA), whereas stars next to the line representing a specific group indicate significant effect of phase within that group, color-coded accordingly (paired *t* test), and black stars below data points indicate significant effect of group (one-way ANOVA). Error bars display SEM throughout. ***P < 0.001, **P < 0.01, *P < 0.05. *N* numbers for animals included in behavioral and electrophysiological analysis are stated in related panels **c** and **h**, **k**, respectively, for each group

Male and female *Gria1*-knockout (*Gria1*^*–/–*^, *Gria1*^tm1Rsp^; MGI:2178057)^25^ mice and WT littermate controls were tested during the light phase of a 12 h light/dark cycle. All experiments conformed to the Animal (Scientific Procedures) Act 1986, UK, and the Local Ethical Review Committee at the University of Oxford. Electrode implantation surgeries and recordings were conducted as previously described^34^. In brief, single polyimide-insulated tungsten wires of 50 µm diameter were implanted into the PFC (AP + 1.7–1.8 mm, ML + 0.25–0.35, 1.7–1.9 mm below pia), dHipp (AP −2 mm, ML + 1.5 mm, −1.3 mm below pia) and ventral hippocampus (vHipp; AP −3.3 mm, ML −2.9 mm, −3.3 mm below pia) of the right hemisphere, whereas a 120 µm reference wire (stainless steel, polytetrafluoroetheylene-insulated) was implanted above the left frontal cortex, and a 1.2 mm ground screw above the cerebellum (Fig. 1f). Recordings from the reference and the vHipp wire were not used for the present analysis.

To reintroduce the AMPA receptor subunit GLUA1 selectively into the hippocampus of *Gria1*-deficient mice, we engineered a recombinant adeno-associated virus of serotype 5 for heterologous expression of an AU1-tagged GLUA1(flip)^35^ mouse isoform under the control of the human synapsin promoter (rAAV-hSyn-AU1-GLUA1; titer of 1.3 × 10^12^ IU/ml: Supplementary Fig.1a). The vector was injected into the entire hippocampus of those *Gria1*^–/–^ mice that constituted the rescue group (GLUA1^CA2/3^) using three infusion sites bilaterally (Supplementary Fig. 1b). Electrode placements were determined post mortem by making electrolytic lesions at the end of experiments and virus-expression was verified using immunohistochemical labeling against GLUA1 and the AU1-tag (Supplementary Figs. 1, 2).

### Behavioral testing and electrophysiological recordings

The T-maze rewarded alternation test of spatial working memory and the Y-maze test of spatial novelty preference were conducted as previously described^34^. In brief, for the T-maze test, animals were food-restricted to 85% of their free-feeding weight. Multiple daily habituation sessions were conducted prior to spatial working memory testing to ensure anxiety-free exploration. Subsequently, animals were trained for 7 days, with 10 trials/day, during which LFPs in dHipp and mPFC were recorded. Each trial consisted of a sample run, in which animals were forced into one pseudo-randomly selected goal arm, and a choice run, in which animals chose freely between both goal arms (Fig. 1a). Animals were rewarded when entering the previously unvisited arm. The Y-maze spatial novelty preference test was conducted once per animal. During the sample phase (5 min), mice could explore the start arm and one goal arm (counter-balanced for group), whereas all arms were accessible during the test phase (5 min, Fig. 5a). Finally, for testing novelty-induced locomotion, animals were allowed to explore clean plastic cages with fresh saw dust freely for 5 min. Locomotor activity was video-tracked in both tests.

Prior to all tests, a 32-channel RHD2132 headstage (Intan Technologies, CA, US) was plugged into the implanted connector via a custom-built adaptor and wired to an open-ephys (MA, USA) acquisition board via two light-weight flexible SPI-cables (Intan Technologies), daisy-chained through a custom-connected miniature slip-ring (Adafruit, NY, US). The adaptor was wired so that all signals were referenced to the ground-signal obtained from above the contralateral cerebellum. Data were amplified and digitized by the RHD2132 headstage, sampled at 15 kHz, and digitally bandpass filtered between 0.1–300 Hz before further processing in MatLab (MathWorks, MA, US) using the open-source Chronux Toolbox. Unless stated otherwise, all data were analyzed with univariate or repeated-measures analysis of variance (ANOVA), as applicable, and Tukey-HSD post hoc tests. See Supplementary Methods for further details on these procedures and analyses.

## Results

### Spatial working memory impairment in *Gria1*-deficient mice can be partially rescued by heterologous GLUA1 expression in hippocampal regions CA2/CA3

Previous studies have suggested a relationship between working memory performance in rodents and both hippocampal–prefrontal theta coherence on the one hand and GLUA1-containing AMPA receptors on the other^24,36–38^. To specifically study the role of hippocampal GLUA1 receptors, we synthesized an rAAV vector to selectively reintroduce GLUA1 into *Gria1*^*–/–*^ mice (Supplementary Fig. 1a). Intra-hippocampal rAAV injection led to successful transduction of AU1-GLUA1 along the longitudinal axis of the hippocampus in GLUA1^CA2/3^ (rescue) mice, but with exclusive expression in the CA2 and CA3 subfields, leaving CA1 and the dentate gyrus uninfected (Supplementary Fig. 1c–d). As the hSyn-promoter of the viral vector could theoretically support expression in all neurons^39,40^, we confirmed the surprising absence of CA1-expression by co-staining for AU1-GLUA1 and markers for CA1 (WFS1^41^) vs. CA2 (PCP4^42^) pyramidal cells (Supplementary Fig. 1e–f), and verified this absence in all injected animals.

We first subjected *Gria1*^–/–^ (*Gria1* knockout), WT (controls), and GLUA1^CA2/3^ (rescue) mice to the T-maze rewarded alternation test of spatial working memory (Fig. 1a). Performance increased across the seven training blocks (*P* = 0.010; repeated-measures ANOVA; see Supplementary Table S1 for statistical details on this and all subsequent analysis, including *F* values; Fig. 1b), improving significantly in the first three blocks (*P* = 0.005). During the last four blocks, performance remained stable (*P* = 0.512) but there was a strong effect of group (*P* = 0.001). This was driven not only by the superior performance of the WT group over the two other groups (*P* < 0.001, Tukey-HSD), but also by a partial, but significant, rescue in the GLUA1^CA2/3^ mice relative to the *Gria1*^–/–^ group (*P* = 0.034; Fig. 1c). This was similar to the behavioral rescue seen previously with broad transgenic restoration of GLUA1 in forebrain excitatory neurons in the same task^43^, which paralleled the restoration of hippocampal synaptic plasticity in these animals^44^. In contrast, *Gria1*^–/–^ mice performed at close to chance levels throughout (Fig. 1b–c), confirming their robust spatial working memory deficit^24,45^.

### In familiar environments hippocampal–prefrontal theta coherence is normal in *Gria1*-deficient mice irrespective of impaired spatial working memory

In a subset of mice, electrophysiological recordings were made from medial PFC and dHipp during spatial working memory testing (Fig. 1f, g). We first analyzed the power of local oscillations in different frequency bands during the last 1.2 s before animals exited the decision zone, during both the sample and choice phases (Fig. 1h–i). This time interval was selected based on median times spent in the “decision zone” (Fig. 1d–e). When combining all three groups, we found that the mean power of local delta (1–4 Hz) and theta (6–12 Hz) oscillations and the peak theta oscillation power in both brain regions, as well as the mean power of beta (20–30 Hz) oscillations in PFC significantly decreased in the choice phase compared with the sample phase (*P* < 0.05, *P* > 0.05 for phase×group interactions, repeated-measures ANOVA, Fig. 1j, k). This indicates, that—in the familiar environment of the T-maze—the power and phase-specific modulation of local delta and theta oscillations are independent of GLUA1 expression. In contrast, dHipp beta and gamma (30–48 Hz) oscillations were significantly modulated by the factor of group in both phases (*P* < 0.05, ANOVA), appearing higher in WT mice compared with the two other groups; however in individual between-group comparisons only trend-level significance was reached (0.05 < *P* < 0.1; Tukey post hoc test; Fig. 1j, k).

**Fig. 2:**
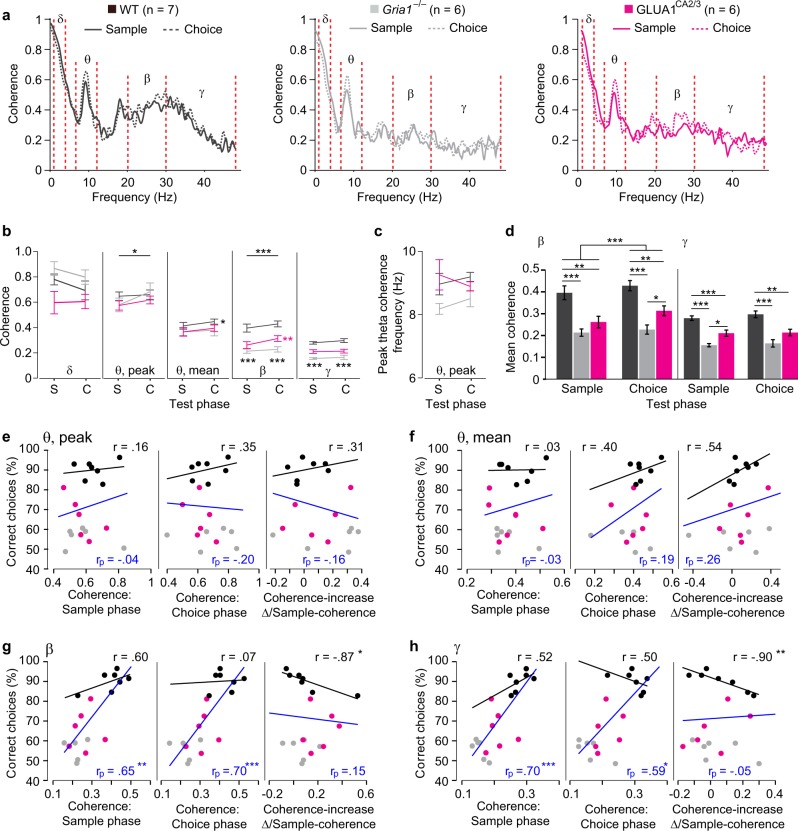
Beta- and gamma rather than theta hippocampal–prefrontal coherence are related to working memory performance. **a** dHipp-PFC coherence plots in WT (left, black), *Gria1*^–/–^ (center, gray), and GLUA1^CA2/3^ (right, magenta) animals. Solid and dashed lines indicate coherence in the sample and choice phase, respectively. Delta (*δ*), theta (*θ*), beta (*β*), and gamma (*γ*) frequency bands used for subsequent analysis are indicated between red-dashed lines (set as in Fig. 1). **b** Quantification of mean delta (*δ*), peak theta (*θ*), mean theta (*θ*), mean beta (*β*), and mean gamma (*γ*) coherence in the sample (S) and choice (C) task phase, as indicated. **c** Frequency of peak theta coherence. **b**, **c**, stars on top line in each subpanel indicate significant effect of test phase across all three groups (repeated-measures ANOVA), whereas stars next to the line representing a specific group indicate significant effect of phase within that group, color-coded accordingly (paired *t* test), and black stars below data points indicate significant effect of group (one-way ANOVA). **d** Quantification of mean beta and gamma coherence, same data as in **b** but depicted to more clearly show group differences within the sample and choice phase. Stars in top line in beta-subpanel show significant effect of phase across groups (repeated-measures ANOVA), whereas stars on lower lines indicate pairwise differences (Tukey post hoc test, conducted after significant effect of group in ANOVA). **e**–**h** Scatter plots depicting peak theta **e**, mean theta **f**, mean beta, **g** and mean gamma, **h** coherence averaged across sample phases (left), choice phases (middle), or expressed as average fractional change ((coherence in choice phase–coherence in sample phase)/coherence in sample phase; right) against average SWM behavioral performance for each animal. Regression lines resulting from multiple linear regression analysis across all groups (blue) or simple linear regression analysis within the wildtype-subgroup (black) are shown for each dataset; in corresponding color-code, the resulting partial (*r*_p_) and normal (*r*) correlation coefficients, indicating the strength and direction of the relationship between coherence and performance are stated, and significance levels of the association between performance and SWM performance are indicated with stars. Note that multiple regression analysis takes prior group differences into account, using both group-identity and coherence as predictors for performance; however, coefficients and significance level for the predictive value of group are omitted for clarity. In all cases **b**–**d** error bars display SEM. ***P < 0.001, **P < 0.01, *P < 0.05. *N* numbers for the subgroups are stated above panel **a**

Next, we analyzed the coherence of oscillations between the dHipp and the PFC (averaged across training blocks, see Supplementary Methods) at various frequency bands, of which theta (6–12 Hz) showed a pronounced peak (Fig. 2a). In contrast to local theta oscillations—and in line with previous studies^36–38^—there was a significant overall increase in the mean theta coherence in the WT group (but not across all groups), and also in peak theta coherence across all groups (but not within any group alone) in the choice phase compared with the sample phase (Fig. 2b). However, for both of those theta coherence measures, there was no significant phase×group interaction and surprisingly, no overall effect of group, regardless if analyzed across phases (repeated-measures ANOVA), or for sample and choice phases individually (univariate ANOVA, Fig. 2b). This indicates that the amplitude of hippocampal–prefrontal theta coherence and its phase-specific modulation were similar between groups, despite the clear spatial working memory deficit in the *Gria1*^–/–^ mice, thus breaking the link between hippocampal–prefrontal theta coherence and spatial working memory performance.

### Hippocampal–prefrontal beta and gamma coherence reflect working memory performance and are partially dependent on GLUA1 in hippocampal subfields CA2/CA3

Similar to theta coherence, mean beta (20–30 Hz) hippocampal–prefrontal coherence was significantly elevated in the choice run compared with the sample run when combining all groups (*P* = 0.001, repeated-measures ANOVA), whereas within individual groups, significance was only reached within the rescue mice (paired *t* test; Fig. 2b, d). The increase in theta and beta coherence in the choice phase is unlikely to simply reflect a general increase in coherence, as we found no significant increase in delta (1–4 Hz) or gamma (30–48 Hz) coherence (Fig. 2b, d). Furthermore, the coherence increase was not simply mediated by an overall increase in local oscillations or volume conductance, as the power of local theta, beta, and delta oscillations in dHipp and mPFC mostly decreased in the choice phase relative to the sample phase (Fig. 1h–k; Supplementary Discussion).

In addition, both beta and gamma coherence during T-maze testing differed between groups across both task phases (*P* < 0.0005, repeated-measures ANOVA). Both WT and GLUA1^CA2/3^ mice showed higher beta and gamma coherence than *Gria1*^–/–^ animals, but also differed between each other (Fig. 2d), indicating a partial rescue that mimicked the partial restoration of behavioral performance (Fig. 1c).

To estimate the relationship between coherence in different frequency bands and T-maze performance, we calculated multiple linear regressions, thereby including coherence and group-identity (accounting for prior group differences) as predictor variables. As the increase of coherence in the choice relative to the sample phase is viewed as an indication for its role in working memory^36^, we also calculated regressions using the relative difference between average choice phase coherence and the average sample phase coherence as a predictor. In contrast to previous assumptions^36,37^, neither peak nor mean theta coherence bore any significant predictive value for T-maze performance, irrespective of whether sample, choice, or sample-to-choice increase were used as a predictor, nor if the regression was calculated across all three groups or only within the WT group (Fig. 2e–f). In contrast, beta and gamma coherence in both phases could significantly predict T-maze performance when including all groups. Also, the coherence increase in both frequency bands could predict working memory scores within the WT group alone (but not across all groups; Fig. 2g–h). Note, however, that this increase was correlated inversely with performance, i.e., the working memory score was lower the lower the average sample phase coherence was relative to the average choice phase coherence. This could indicate that very high beta/gamma coherence is already needed in the sample phase (and then stays high into the choice phase) to enable high working memory performance. As an additional note, in all regression models calculated across all three groups the variable of group could predict performance with at least trend-level (*P* < 0.1) and mostly significant (*P* < 0.05) predictive power.

### GLUA1 reintroduction to area CA2/CA3 rescues hyperactivity in *Gria1*^–/–^ mice

Previous studies have also demonstrated an important role for GLUA1 in short-term habituation^23,26^. Exploratory drive is related to the extent to which an environment is perceived as interesting owing to the fact that there are unfamiliar and therefore salient spatial stimuli that attract attention. A decrease in exploration, indicated by reduced locomotor activity over time, is reliably observed when rodents familiarize themselves with novel environments, reflecting, at least in part, short-term habituation to environmental cues.

**Fig. 3:**
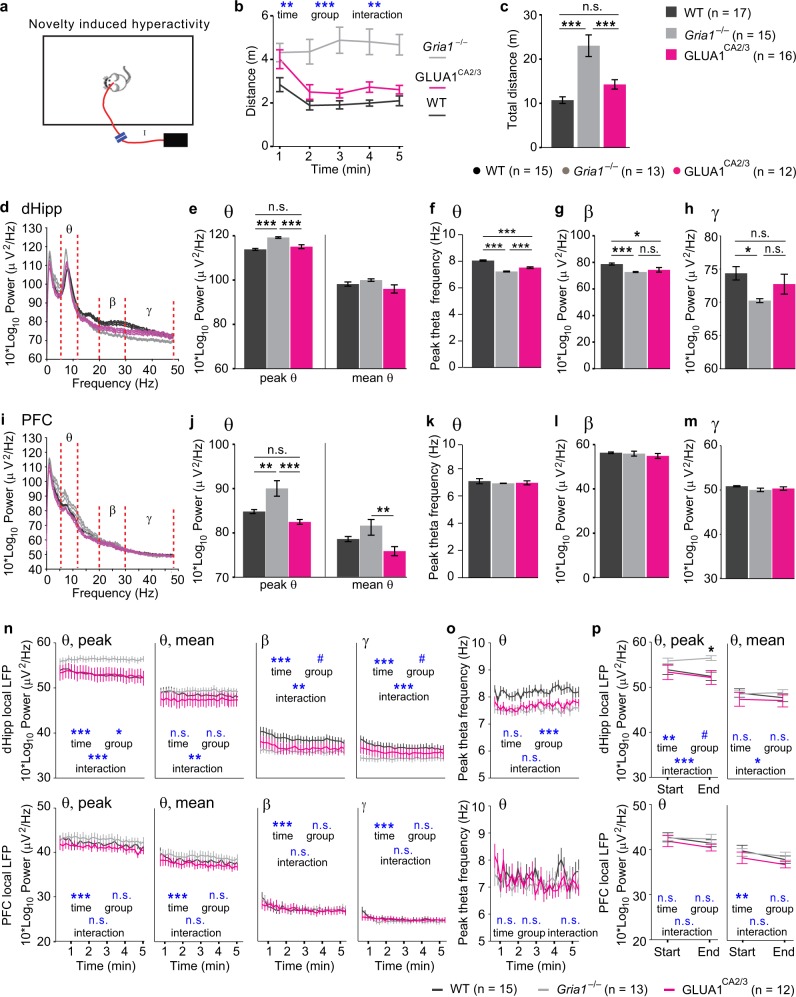
Decreased theta power reflects short-term habituation in the open field. **a** Schematic of novel environment recording setup. **b** Minute by minute locomotion, and **c** total distance traveled by WT (black), *Gria1*^–/–^ (gray) and GLUA1^CA2/3^ (magenta) animals during novel environment exploration. Stars in **c** indicate pairwise Tukey post-hoc tests conducted after identifying a significant effect of group (*P* < 0.001; repeated-measures ANOVA over time-bins). **d**, **i** Group-average power spectra ± SEM in the dHipp **d** or PFC **i** with theta (*θ*, 6–12 Hz), beta (*β*, 20–30 Hz), and gamma (*γ*, 30–48 Hz) frequency ranges shown between red dotted lines. **e**, **j** Average peak theta (left) and mean theta (right) power in dHipp **e** or PFC **j**. **f**, **k** Frequency of the peak theta frequency in dHipp **f** or PFC **k**. **g**, **h**, **l**, **m** Average beta **g** (dHipp), **l** (PFC) and gamma **h** (dHipp), **m** (PFC) power. **n** Average power of local peak theta (*θ*), mean theta (*θ*), beta (*β*), and low-gamma (*γ*) oscillations in dorsal hippocampus (top) and prefrontal cortex (bottom), as indicated, in 10 s-bins across the 5 min testing period in the open field, shown for WT (black), *Gria1*^–/–^(gray), and GLUA1^CA2/3^ (magenta) groups. **o** Same display as in **n** but indicating average peak frequency of theta oscillations. **p** Same data as in **n**, *θ* but displaying peak and mean theta power only for the first and the last 10 s interval of the exploration period. Blue symbols in **b**, **n**–**p** show significance level of effects of time, group, and group×time interaction (as indicated) obtained in repeated-measures ANOVAs over the shown 5 **b**, 30 **n**, **o**, or 2 **p** time-bins). Black stars in **p** indicate effect of group at the respective time point (one-way ANOVA). Black stars in bar graphs (**c**, **e**–**h**, **j**–**m**) indicate results of pairwise Tukey post hoc tests if a significant effect of group was found in overall ANOVA. ***P < 0.001, **P < 0.01, *P < 0.05, ^#^*P* < 0.1, n.s. *P* > 0.1. In all cases error bars display the SEM. *N* numbers for animals included in behavioral and electrophysiological analysis are stated in related panels **c** and above **f**–**h**, respectively, for each group

As shown previously^20,27,28^, *Gria1*^–/–^ animals displayed sustained and prolonged hyperactivity during exploration of a novel environment (Fig. 3a–b, repeated-measures ANOVA over five 1-min-bins: *P* *<* 0.001 for effects of time-bin, group and interaction). *Gria1*^–/–^ mice were markedly hyperactive compared with both WT mice and also GLUA1^CA2/3^ animals (*P* *=* 0.001), with no significant difference between the latter two groups (*P* *=* 0.227; Fig. 3c), suggesting a robust rescue of short-term habituation by GLUA1 re-introduction into hippocampal CA2/CA3, as seen before in a similar study with GLUA1 re-expression in all subfields of the hippocampus^40^.

The behavioral pattern observed in this test–including differences between the three groups and changes over time—provided us with a blueprint to identify electrophysiological correlates of short-term habituation of attention. Such electrophysiological correlates should recapitulate the behavior in the sense that: (a) *Gria1*^–/–^ animals should be distinct from WT controls, (b) reconstitution of GLUA1 in CA2/CA3 should rescue any aberration, i.e. GLUA1^CA2/3^ mice should be distinct from knockouts and similar to WT animals, and (c) these differences should be most marked towards the end of the testing period, driven by a change of the measure over time in WT controls and rescue animals, compared with knockouts (divergence). Statistically this pattern should be highlighted by significant effects of group and a group×time interaction.

### Elevated and unmodulated local theta oscillations in *Gria1*^–/–^ animals in a novel environment are rescued by GLUA1 reintroduction into CA2/CA3

During the exploration of a novel environment, the peak theta frequency power of the LFP oscillations in both the dHipp and the mPFC were significantly elevated in *Gria1*^–/–^ mice compared with both WT and GLUA1^CA2/3^ animals (*P* *<* 0.005), whereas these latter two groups were indistinguishable from each other (*P* *>* *0.2*; Fig. 3d–f, i–k). This suggests that not only hippocampal, but even prefrontal theta oscillations can be modulated by hippocampal GLUA1-containing AMPA receptors in CA2/CA3. Mean theta power, in contrast, was largely unaffected (Fig. 3e, j), suggesting that the increase in peak theta frequency power was mediated by a narrow, frequency-stable and dominating oscillation. In addition, the peak frequency of dHipp theta oscillations was significantly lower in *Gria1*^–/–^ animals compared with both the other groups, whereas GLUA1^CA2/3^ mice showed a partial rescue of this parameter (Fig. 3f).

In contrast to theta oscillations, beta oscillations in the dHipp displayed significantly higher power in WT mice compared with both *Gria1*^–/–^ (*P* *=* 0.001) and GLUA1^CA2/3^ (*P* *=* 0.01) animals, which showed similar power (*P* *=* 0.564; Fig. 3g), indicating the lack of a rescue effect. Beta oscillations in the PFC did not differ between groups (*P* = 0.405; Fig. 3l). A similar pattern was seen for local gamma oscillations (Fig. 3h, m).

In order to further evaluate the potential link between dHipp and PFC network activity, GLUA1 expression and short-term habituation, we analyzed these electrophysiological measures over the time course of the locomotor experiment (Fig. 3n–o). Although peak theta frequency and the power of local oscillations in various frequency bands showed a significant decrease over time, only dHipp peak theta power displayed an additional divergence between knockouts and the two other groups, resembling the behavioral readout of short-term habituation (see Fig. 3b): peak theta power remained elevated in *Gria1*^–/–^ mice throughout the session, whereas in the two other groups it decreased leading to significant a effect of group (*P* < 0.05) and a group×time interaction (*P* < 0.001, repeated-measures ANOVA; Fig. 3n–o). This conclusion was confirmed when analyzing only the first vs. the last 10 s intervals of the testing period, in which novelty-related attention is predicted to be most different (Fig. 3p). In contrast, dHipp beta and gamma power were higher in the WT and rescue groups and then decreased towards the lower power seen in the knockout group (which was stable over time), yielding significant effects of time and interactions, but only trend-level effects of group (Fig. 3n).

**Fig. 4:**
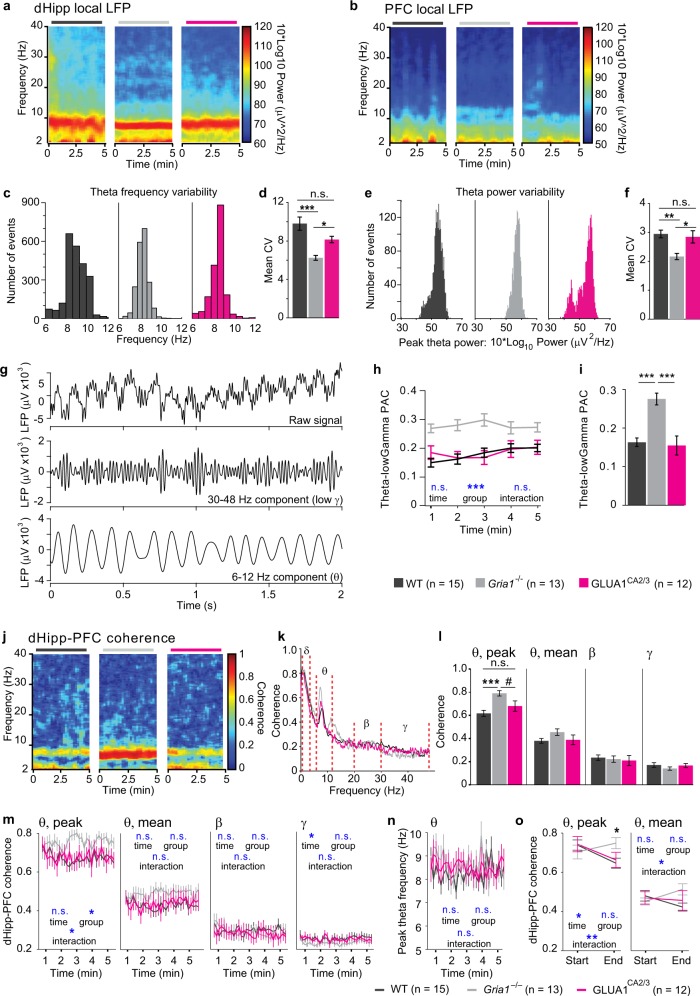
Hippocampal GLUA1-dependence of local and long-range oscillatory dynamics during short-term habituation in the open field. **a**, **b** Representative examples for power spectrograms in dHipp **a** and PFC **b** for WT (left), *Gria1*^–/–^ (middle), and GLUA1^CA2/3^ (right). **c**, **e** Histograms plotting the distribution of values of dHipp peak theta frequency **c** and power **e** calculated for each animal in individual non-overlapping 2 s bins. **d**, **f** Quantification of the coefficient of variance (CV) of the data shown in **c**, **e** for theta frequency **d** and theta power **f**. Symbols above bars (*, n.s.) indicate results from pairwise Tukey-HSD post hoc tests conducted after finding a significant effect of group in the ANOVA (*p* ≤ 0.002) for both parameters. **g** Example LFP traces with raw data (top), gamma-range filtered (middle) and theta-range filtered (bottom) components. **h** Phase-amplitude coupling (PAC) of theta-gamma oscillations over time (1-minute bins). **i** Quantification of PAC collapsed over time. **j** Representative examples of dHipp-PFC coherograms for WT (left), *Gria1*^–/–^ (middle), and GLUA1^CA2/3^ (right). **k** Average dHipp-PFC coherence plots with delta (*δ*, 1–4 Hz), theta (*θ*, 6–12 Hz), beta (*β*, 20–30 Hz), and gamma (*γ*, 30–48 Hz) frequency bands indicated between red dashed lines. **l** Average peak theta, mean theta, mean beta, and mean gamma dHipp-PFC coherence, as indicated. **m** Average peak theta, mean theta, mean beta, and mean gamma dHipp-PFC coherence over time (in 10 s bins). **n** Same display as in **m** but indicating average peak frequency of theta oscillations. **o** Same data as in **m** (*θ*) but displaying peak and mean theta power only for the first and the last 10 s interval of the exploration period. Blue symbols in **h**, **m**–**o** show significance level of effects of time, group, and group×time interaction (as indicated) obtained in repeated-measures ANOVAs over the shown 5 **h**, 30 **m**, **n**, or 2 **o** time-bins). Black star in **o** indicates effect of group at the respective time point (one-way ANOVA). Black stars in bar graphs **d**, **f**, **i**, **l** indicate results of pairwise Tukey post hoc tests if a significant effect of group was found in overall ANOVA. ***P < 0.001, **P < 0.01, *P < 0.05, ^#^*P* < 0.1, n.s. *P* > 0.1. In all cases error bars display the SEM. *N* numbers for each group are stated at the bottom of the figure

In line with an increase of theta oscillations at a narrow peak frequency, instead of broadly across the 6–12 Hz-band (mean theta power, see above), we also noted that dHipp theta oscillations seemed to look less variable in individual spectrograms of *Gria1*^–/–^ mice compared with the other groups (Fig. 4a). Analyzing the variation of peak frequency and peak power of dHipp theta oscillations over time, we found that both measures were indeed less variable in knockouts, while normal levels of variability were largely restored by re-introduction of GLUA1 in CA2/CA3 (Fig. 4c–f).

Given the aberrations of dHipp theta and gamma oscillations, we next analyzed phase-amplitude coupling (PAC) between these two oscillatory components^46^. We found that theta-gamma PAC was strongly elevated in *Gria1*^–/–^ animals compared with the two other groups (*P* *<* 0.001, ANOVA), which, in turn, were indistinguishable from one another (Fig. 4g–i). However, the groups qualitatively converged—rather than diverged—over the course of the experiment, suggesting that it is unlikely that this measure reflects short-term habituation (Fig. 4h).

In summary, GLUA1-containing AMPA receptors are essential to maintain normal levels of theta, beta, and gamma oscillations in the dHipp and to regulate frequency and variability of hippocampal theta oscillations and theta-gamma PAC. Surprisingly, reconstitution of GLUA1 expression in CA2/CA3 alone was sufficient to restore normal levels of power and variability of dHipp theta oscillations, theta-gamma PAC, and even the power of PFC theta oscillations in Gria1-knockout animals. However, of all these local measures, only the peak power of dHipp theta oscillations showed a pattern that fully resembled short-term habituation of attention at the behavioral level in our cohort.

### Elevated and unmodulated hippocampal–prefrontal theta coherence in *Gria1*^–/–^ animals in a novel environment is rescued by GLUA1 reintroduction into CA2/CA3

Strikingly, when compared with WT mice, *Gria1*^–/–^ mice also showed strongly *elevated* peak theta coherence between dHipp and PFC during the exploration of a novel environment (*P* = 0.001 for ANOVA across groups and for WT versus *Gria1*^–/–^ comparison; Fig. 4j–l). GLUA1^CA2/3^ animals showed a partial reduction of this elevated coherence, being numerically lower than in *Gria1*^–/–^ mice (*P* *=* 0.066) and statistically similar to WT mice (*P* *=* 0.260; Fig. 4l). This was specific to the theta band, as there were no significant differences in the mean delta, beta, or gamma coherence (*P* > 0.4; Fig. 4l, Supplementary Fig. 3a).

When analyzing peak theta coherence across the complete 5-min exploration period in 10 s bins, we found a significant effect of group (*P* *=* 0.047) and a group×time interaction (*P* *=* 0.007), owing to the overall decrease in the combined WT and GLUA1^CA2/3^ groups (*P* *=* 0.002) and an actual increase in theta coherence in *Gria1*^–/–^ animals across the exploration period (*P* = 0.011, repeated-measures ANOVA; Fig. 4m). In contrast, no relevant statistical effects were seen for mean theta, beta or gamma coherence, nor for the peak frequency of theta coherence (Fig. 4m–o). In addition, we compared dHipp-PFC peak theta coherence during the first and the last 10 s of the 5-min exploration period, when familiarity of the context is expected to be most different. Again, there was a significant interaction between group and time-period (*P* *=* 0.008), driven by the predicted decrease of theta coherence in the WT and GLUA1^CA2/3^ groups (*P* < 0.05, Bonferroni paired comparison) and an absence of this decrease in knockouts (*P* *=* 0.180; Fig. 4o). As seen with local dHipp theta power, the divergence of dHipp-PFC theta coherence between groups over time led to a significant effect of group in the last 10 s interval, whereas no such difference was observable in the first interval, as predicted from the behavioral deficit in short-term habituation (Fig. 4o).

These data differ markedly from the recordings made in the T-maze (Fig. 1h, j and Fig. 2a–b,) where local theta power and theta coherence were indistinguishable between groups. We speculated that these differences relate to the fact that the environment used for assessing locomotor activity was novel, whereas the T-maze environment was highly familiar by the time spatial working memory testing started owing to the extensive habituation of the mice to the maze environment prior to testing (note that long-term habituation is intact in *Gria1*^–/–^ mice^26^). Therefore, the elevated peak theta coherence and dHipp peak theta power could reflect the levels of selective attention induced here by spatial novelty and the deficit in short-term habituation in *Gria1*^–/–^ mice.

### GLUA1 in CA2/CA3 is necessary for spatial novelty preference

**Fig. 5:**
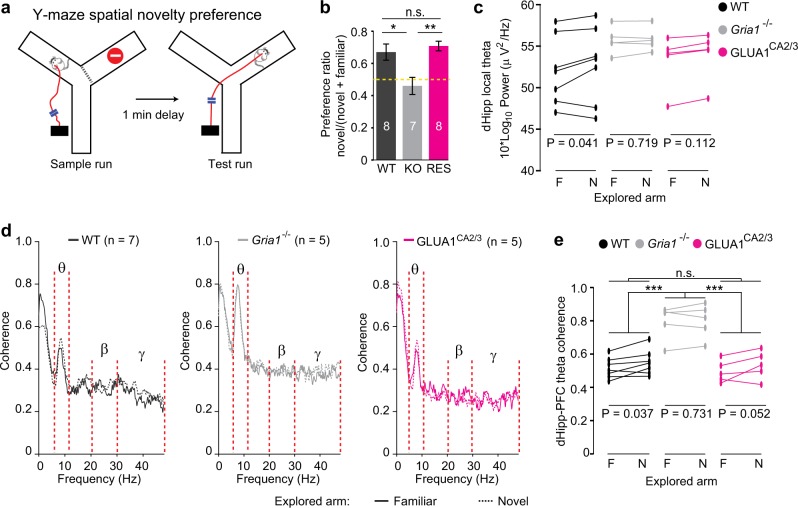
Theta power and coherence reflect relative spatial novelty in the Y-maze. **a** Schematic of spatial novelty preference (SNP) Y-maze task. **b** Novel arm preference ratio (time spent in novel goal arm divided by time spent in both goal arms) of WT (black), *Gria1*^–/–^ (gray), and GLUA1^CA2/3^ (magenta) animals. Yellow dotted line indicates chance level performance. Stars indicate pairwise Tukey's post hoc tests conducted after a significant effect of group was found (*P* = 0.002; univariate ANOVA). *N* numbers are indicated within bars and error bars show SEM. **c** Average peak theta power in dorsal hippocampus during bouts exploring the familiar (F) vs. novel (N) Y-maze arm shown for each animal. **d** Coherence plots for bouts of exploration in the familiar (solid lines) versus novel (dashed lines) arm of the Y-maze during the test phase; with key frequency bands indicated (see also Supplementary Fig. 4). **e** Average peak theta frequency hippocampal–prefrontal coherence during bouts exploring the familiar (F) versus novel (N) Y-maze arm, shown for each animal. For **c**, **e** results of pairwise comparisons between groups (LSD post hoc test) are indicated above top lines, if a significant effect of group was found (repeated-measures ANOVA), whereas results of paired *t* tests within groups, comparing novel vs. familiar arm are indicated at the bottom of each graph. ***P < 0.001, **P < 0.01, *P < 0.05, n.s. *P* > 0.05. *N* numbers for animals used for electrophysiological analysis are indicated in **d**

To directly test the hypothesis that theta coherence can reflect spatial novelty-related attention, we turned to a paradigm, which allows within-task comparisons between exposure to novel versus familiar spatial stimuli, namely the SNP Y-maze test. This task assesses a form of short-term memory that underlies stimulus-specific short-term habituation and is dependent on both the hippocampus and GLUA1^22,26,47^. Animals initially familiarize themselves with both the start arm and one of the goal arms of the maze (sample phase), so that in the test phase they are exposed to both a more familiar spatial stimulus when they visit the familiar arm, and to a novel spatial stimulus when they explore the unfamiliar arm (Fig. 5a).

The preference for the novel arm (preference ratio = (time in novel goal arm)/(time in both goal arms)) during the first 1 min of the test phase was different between groups (*P* *=* 0.002; ANOVA), whereas WT and GLUA1^CA2/3^ mice showed a similarly strong preference for the novel arm (*P* *=* 0.819; Tukey post hoc test), *Gria1*^–/–^ mice performed at chance levels (0.5; *P* < 0.02 for differences between knockouts and the other two groups, Fig. 5b). This indicates that short-term spatial novelty preference is fully impaired by global *Gria1* knockout^26,47^, but then completely rescued by GLUA1 reintroduction into hippocampal CA2/CA3.

### Hippocampal–prefrontal theta coherence tracks habituation to spatial novelty in WT and GLUA1^CA2/3^ rescue animals but remains elevated in *Gria1*^–/–^ mice

We recorded LFPs during the spatial novelty preference Y-maze test and focused our analysis on the two electrophysiological signatures that appeared to reflect novelty-induced attention and its decrease due to short-term habituation in the prior open-field test: local dHipp peak theta power and dHipp-PFC peak theta coherence. We calculated each measure during bouts of exploration in the novel and familiar arms during the test phase. As predicted, we found that there was a small, but statistically significant increase in the local dHipp peak theta power during visits to the novel versus the familiar arm (*P* *=* 0.031, repeated-measures ANOVA). This appeared to be driven primarily by WT mice (Fig. 5c), although there was no significant group×arm interaction (*P* *=* 0.489; repeated-measures ANOVA). However, in contrast to the prior exploration test in the open field, there was no significant effect of group on the levels of theta power in the test phase of the Y-maze, neither when combining the episodes spent in the novel and familiar arm (*P* *=* 0.270, repeated-measures ANOVA) nor when analyzing only the periods when the mice were in the familiar arm, where the strongest difference is predicted given the prior exposure to that same area during the sample phase (*P* *=* 0.353, ANOVA).

In contrast, hippocampal–prefrontal peak theta coherence mirrored the group differences seen behaviorally during the Y-maze task and fully conformed to our prior predictions: regardless of arm, *Gria1*^–/–^ mice generally displayed strongly increased peak theta coherence (*P* < 0.001 for post hoc comparisons between WT and *Gria1*^–/–^), whereas theta coherence in GLUA1^CA2/3^ mice was again reduced to WT levels (*P* *=* 0.92; Fig. 5d, e). In addition, the peak theta coherence was significantly higher, whereas mice were exploring the novel arm compared to the familiar arm (*P* *=* 0.018, within-subject comparison; Fig. 5e). Also, this novelty-induced increase in coherence was specific to peak theta coherence, as it was not detectable when analyzing mean theta, beta, and gamma coherence or PFC local peak theta power (Supplementary Fig. S4). Qualitatively, this increase in theta coherence was only present in the WT and GLUA1^CA2/3^ mice, and not in *Gria1*^–/–^ animals (see Fig. 5e), although there was no significant group×arm (i.e., novel versus familiar) interaction (*P* *=* 0.393).

Also, importantly, there was no effect of arm (novel vs. familiar) on running speeds (data not shown; *P* *=* 0.491), suggesting that differences in coherence between novel and familiar arms did not simply reflect motor activity. As expected, *Gria1*^–/–^ mice showed elevated running speeds compared with both WT and GLUA1^CA2/3^ animals (*P* < 0.002 for each group, data not shown).

## Discussion

In this study, we measured local oscillations and long-range coherence between the hippocampus and PFC of *Gria1*^–/–^ and WT mice while they performed three hippocampus-dependent tasks assessing spatial forms of short-term memory and attentional processing^19–21,48,49^. We found that (i) peak theta coherence and dHipp peak theta power were increased during behavioral epochs in which general levels of attention were elevated owing to spatial novelty, (ii) GLUA1 expression in CA2/CA3 modulated these behavioral and physiological processes, and (iii) beta- and gamma- (but not theta-) coherence related to working memory performance on the rewarded alternation T-maze task. These findings have implications for understanding the physiological basis of working memory and selective attention, as well as for the pathophysiology underlying the causal link between schizophrenia and its putative risk gene *GRIA1*.

### Hippocampal–prefrontal theta coherence reflects novelty-induced selective attention and is regulated by GLUA1 in CA2/CA3

*Gria1*^–/–^ knockout mice exhibited a pronounced deficit in short-term habituation, which was reflected by exaggerated levels of exploration in a novel environment and by a deficit on the spatial novelty preference test, both of which were largely rescued by the re-introduction of GLUA1 into CA2/CA3. The pattern of group differences and time-dependent effects seen in those two behavioral tasks allowed us to search for electrophysiological correlates of short-term habituation of stimulus-specific attention provoked by spatial novelty. Our analysis of a wide range of electrophysiological readouts, including hippocampal–prefrontal coherence and the power of local oscillations in four frequency bands revealed that—although a surprisingly large number of these parameters were dependent on functional GLUA1 expression in CA2/CA3 (see Results)—only the amplitude of dHipp-PFC peak theta coherence could be reproducibly linked to spatial novelty-induced attention and short-term habituation.

Accordingly, during spatial working memory testing in the highly familiar T-maze environment, theta frequency hippocampal–prefrontal coherence largely appeared normal in *Gria1*^–/–^ animals. This shows that the *Gria1*-knockout-induced increase in local hippocampal peak theta power and peak theta coherence seen in the open field and SNP tests are state-dependent effects (rather than intrinsic changes resulting from GLUA1-ablation) and are determined by the relative novelty/familiarity of the environment. Interestingly, this recapitulates the endophenotype of elevated theta oscillations and theta coherence seen in patients with schizophrenia^8,9,11^, and has also been shown in another mouse model of reduced glutamatergic function (*Sp4* hypomorphs)^50^, which also has an attentional deficit.^51^

### Theta coherence does not reflect T-maze working memory performance

Our observation that theta coherence was normal despite the consistent and complete failure of *Gria1*^–/–^ mice on the T-maze working memory task was surprising and suggests that theta coherence may not provide a readout of intact memory retrieval. Indeed, our regression analyses both across the whole cohort as well as within the WT group did not reveal any obvious link between T-maze performance and theta coherence or theta coherence increase during memory retrieval. Previous studies assessing spatial working memory in rats^36^ and mice^37,38^ on the T-maze have found that hippocampal–prefrontal coherence in the theta frequency-range was elevated specifically during the choice run (i.e., when memory contents are retrieved), and that working memory performance correlated positively with pre-training theta coherence in a mouse model of schizophrenia (although, notably, this was not the case in the WT mice in this study when analyzed alone)^37^. This was taken as an indication that theta coherence might represent spatial working memory (SWM) retrieval processes^36–38^. Another possibility, however, is that increased theta coherence might reflect the elevated levels of attention in the choice run^36^. Our data support this alternative hypothesis.

### Beta- and gamma-frequency coherence are dependent on GLUA1 and reflect working memory performance

In contrast to theta coherence, both beta- and low-gamma-frequency hippocampal–prefrontal coherence could significantly predict spatial working performance in regression models calculated across groups. As this predictive power holds for coherence in both the sample and the choice phase, its concrete mechanistic link to working memory remains elusive. For example, although there is an increase of beta coherence in the choice phase relative to the sample phase when analyzing all groups, in WT mice alone this effect did not reach significance and the increase was even negatively correlated with performance. This might reflect the particular importance of beta/gamma hippocampal–prefrontal coherence during the encoding phase of working memory, as recently suggested^52^. Importantly, beta-frequency coherence, albeit between medio–dorsal thalamus and PFC, has previously been related to T-maze spatial working memory performance in mice^53^, and might be a correlate of top–down signaling^54^. However, further experiments will be required to explore the necessity of beta coherence for SWM performance.

### GLUA1 in CA2/CA3 orchestrates hippocampal–prefrontal coherence and local theta oscillations

Beyond those implications for the physiological underpinnings of working memory and spatial novelty-induced attention, our data also reveal a mechanistic link between the putative schizophrenia risk gene *GRIA1*^15,16^ and cognitive deficits in schizophrenia. This is because *GRIA1*-mRNA expression is indeed reduced in CA3 in schizophrenic patients^17,18^, and CA3 has a key role in regulating theta oscillations, as it receives parvalbumin-positive projections from the medial septum^55^, a known modulator of hippocampal theta^56^. Our data imply that reduced expression of GLUA1 in CA3 would be sufficient to cause elevated theta and reduced beta coherence, and thereby result in deficits of selective attention and working memory, respectively.

Previously, a combined electrophysiology-functional MRI study in healthy carriers of a genetic schizophrenia risk factor concluded that hippocampal theta oscillations orchestrate hippocampal–prefrontal co-activation under baseline conditions^7^. Also, a recent study in mice demonstrated that hippocampal–prefrontal theta coherence can be weakened by inhibiting hippocampal, but not prefrontal, interneurons, thereby impairing sustained attention^57^. Our data support this notion because the local rescue of GLUA1 expression in CA2/CA3 restored not only the level and variability of hippocampal theta oscillations, but also hippocampal–prefrontal theta coherence, and—in the case when it was altered—even local prefrontal theta oscillations.

### CA3 as a comparator to control salience attribution

The data presented in this study supports the view that a key function of the hippocampus is to act as a comparator^58–60^. In this view, synaptic plasticity in CA3 may be a key mechanism underpinning short-term habituation of attention, and theta oscillations—induced by novelty—protect the attentional focus from interference. Our data conform to two key predictions of this model: (i) a higher amplitude and smaller variability of dorsal–hippocampal theta oscillations would translate into an elevated and less flexible attentional focus, and (ii) short-term habituation would be impaired due to disturbance of synaptic plasticity by abolishment of GLUA1-containing AMPA receptors. GLUA1 has an important role especially in a short-lasting phase of hippocampal synaptic plasticity^25,61–65^, including for long-term potentiation of recurrent connections between CA3 pyramidal neurons^66^. Both mechanisms (i and ii) would equate to a state, in which sensory stimuli retain high salience despite their extended presence—a key characteristic of aberrant salience in schizophrenia^67,68^. It needs to be noted though, that the original model^59^ assumed a role for plasticity at the incoming mossy-fiber-CA3 synapses carrying cortical input, which is, however, GLUA1-independent. Furthermore, a role of GLUA1-containing AMPA receptors in CA2, mediating LTP in a direct input from the enthorhinal cortex onto distal dendrites of CA2 pyramidal cells^69^ cannot be excluded either.

## Conclusion

Our results suggest that the sustained elevation of hippocampal–prefrontal theta coherence in *Gria1*^–/–^ mice reflects excessive and prolonged levels of selective attention in novel environments, which are unrelated to the actual relevance of stimuli, and which reflect a failure of short-term habituation in these mice. These deficits in habituation, leading to continued and inappropriately high levels of attention, could result in the aberrant assignment of salience to stimuli, a possible driver of psychosis in schizophrenic patients^13,68^. More specifically, our data point to the AMPAR signaling network in hippocampal areas CA2/CA3 as a potential therapeutic target in psychosis.

## Supplementary information

Supplementary Information.
