# Supplementary material for: Hippocampal–prefrontal coherence mediates working memory and selective attention at distinct frequency bands and provides a causal link between schizophrenia and its risk gene GRIA1
Source: Transl Psychiatry. 2019 Apr 18;9:142. doi: 10.1038/s41398-019-0471-0 (PMC6472369; doi:10.1038/s41398-019-0471-0)
Supplement: Supplementary file 1 — Supplementary Information. [file 41398_2019_471_MOESM1_ESM.docx]

# Supplementary Information

Hippocampal-prefrontal coherence mediates working memory and selective attention at distinct frequency bands and provides a causal link between schizophrenia and its risk gene *GRIA1*

***Running title:* GLUA1 AMPA receptors of CA2/3 in aberrant salience**

Alexei M Bygrave ^1,4,6^, Thomas Jahans-Price ^1^, Amy R Wolff ^1,4^, Rolf Sprengel ^2,3^, Dimitri M Kullmann ^4,7^, David M Bannerman ^1,7,8^, Dennis Kätzel ^1,4,5,7,8^

^1^Department of Experimental Psychology, University of Oxford, Oxford, UK

^2^Max-Planck Institute for Medical Research, Heidelberg, Germany

^3^Institute for Anatomy and Cell Biology, Heidelberg University, Germany

^4^Institute of Neurology, University College London, London, UK

^5^Institute of Applied Physiology, Ulm University, Ulm, Germany

^6^Current address: Department of Neuroscience, Johns Hopkins University, USA

^7^These authors contributed equally to this work

**^8^ Correspondence:**

David M. Bannerman, Dept. of Experimental Psychology, University of Oxford, Parks Road, OX1 3PJ, Oxford, United Kingdom, david.bannerman@psy.ox.ac.uk

Dennis Kätzel, Institute of Applied Physiology, Ulm University, 89081 Ulm, Germany; dennis.kaetzel@uni-ulm.de

**SUPPLEMENTARY METHODS**

**Animal subjects**

Adult male and female *Gria1*^–/–^ (Gria1^tm1Rsp^; MGI:2178057) ^1^ and wildtype littermate control mice (C57BL/6 background) were used for experiments. The sample size was chosen based on our prior knowledge of effect sizes for the differences between Gria-knockout, wildtype and (different kind of) rescue animals in the three behavioural tests used in the present study (see below)^2–7^. Animals were housed in a vivarium with a 12 hr light/dark cycle (lights on at 07:00 a.m. and off at 07:00 p.m.). All behavioral testing was conducted during the light phase. All experiments conformed to the Animal (Scientific Procedures) Act 1986, UK, and the Local Ethical Review Committee at the University of Oxford.

**Virus generation**

The DNA sequence of the GLUA1(flip) isoform from the mouse (transcript variant 1) was obtained from GenBank (NCBI, accession number NM_001113325.2) and modified to contain Not1 and Sal1 restriction enzyme sites upstream and downstream of the GLUA1 construct, respectively. To enable antibody-mediated identification of recombinant GLUA1 expression, an AU1 immuno-tag ^8^ was added after the first 18 N-terminal codons of the *Gria1* cDNA that encode the signal peptide for transmembrane localization. The AU1-*Gria1*-encoding DNA fragment was synthesized *de novo* and inserted into NotI/SalI a pAAV-hSyn-*Not1-Sal1*-WPRE-hGHpA vector backbone. Both, insert and backbone were synthesized by GenScript (China). Plasmid DNA was amplified using an Endotoxin-free Maxi-prep kit (Qiagen). Viral DNA encoded by pAAV-hSyn-AU1-GLUA1 was packaged into adeno-associated viral vector serotype 5 (AAV5) capsids by the Vector Core facility of the University of North Carolina (UNC, NC, US). Recombinant AAV particles were suspended in 5 % sorbitol / PBS at a titre of 1.3 x 10^12^ iu/ml). rAAV aliquots stored at -80^o^C for long-term, and, after thawing, at -4^o^C for a maximum of 2 weeks.

**Surgery**

Surgical procedures and implantation of electrodes were as previously described ^9^. Briefly, animals were anaesthetized with isoflurane, given analgesia (meloxicam, buprenorphine and lidocaine) and placed into a stereotaxic frame (David Kopf instruments). Three craniotomies per hemisphere were made above the virus injection and electrode sites. Only the *Gria1*^–/–^ mice used for generation of GLUA1^CA2/3^ mice received such craniotomies in both hemispheres as well as infusions of the undiluted rAAV5-hSyn-AU1-GLUA1 suspension via a Hamilton syringe (34g beveled needle) controlled by a digital injector pump (WPI Instruments) at a rate of 100 nl/min using the following hippocampal coordinates:

| AP coordinate: | ML coordinate: | Z coordinates:  (from brain surface) | Injection volumes (nl) |
| --- | --- | --- | --- |
| -2.2 | +/- 1.8 | -1.5, -1.1 | 400 and 600 |
| -2.8 | +/- 3 | -2, -1.5 | 500 (at each) |
| -3.3 | +/- 3 | -3.5, -2.5 | 500 (at each) |

In all groups, single straightened polyimide-insulated tungsten wires of 50 µm diameter (WireTronic Inc., CA, US) were implanted into the prefrontal cortex (PFC; AP +1.7-1.8 mm, ML +0.25-0.35, 1.7-1.9 mm below pia), dorsal hippocampus (dHipp; AP -2 mm, ML + 1.5 mm, -1.3 mm below pia) and ventral hippocampus (vHipp; AP -3.3 mm, ML -2.9 mm, -3.3 mm below pia) of the right hemisphere and affixed in place with blue light-cured Breeze® self-adhesive resin cement (Pentron, US). Three further craniotomies were made above the left hemisphere for a reference wire (at AP -0.5 mm and ML -1 mm, 125 μm diameter, PTFE insulated, Advent Research Materials Ltd., UK), a ground screw (above the cerebellum,1.2 mm diameter, Precision Technology Supplies Ltd., UK) and an anchor screw (at AP -2 mm and ML -2 mm, details as for ground screw).

**Histology**

Animals were transcardially perfused with PBS followed by 4% paraformaldehyde (PFA)/PBS and brains were post-fixed for 24 h in PFA. Coronal sections of 60 µm were cut on a vibratome in PBS and then washed 3 times in PBS. Sections were incubated in 0.15% TBS-Triton-X (TBST) for 20 min, then 20% horse serum/TBS-T for 1 h, then slices were briefly washed in TBS-T and then incubated with the primary antibodies (rabbit-anti-GLUA1, 1:500, Chemicon, #05855R; rabbit-anti-WFS1, 1:500, Proteintech, #11558-1-AP; rabbit-anti-PCP4, 1:1000; Santa Cruz Biotech (now discontinued); mouse-anti-AU1, 1:500, Covance, #901901) in 2.5% horse serum-TBS-T for 48 h. Following primary antibody incubation, the sections were washed 3 times in TBS-T and then incubated overnight with the fluorescent secondary antibodies (goat Alexa 488 and 568 conjugated; Molecular Probes) diluted 1:500 in 2.5% horse serum/TBS-T containing DAPI. Sections were then washed 3 times with PBS, once in PB and mounted with Vectashield (Vector Labs). Antibody incubations were done at 4^o^C and all other incubations at room temperature. Images were acquired with a fluorescent microscope (Axio Zoom, Carl Zeiss, or DM6, Leica).

**Behavioral testing**

In total, 2 cohorts of *Gria1*^–/–^*,* GLUA1^CA2/3^ and WT littermates each were tested. After virus injection mice were left for 4 weeks for recovery and to give time for virus expression. Mice were tested for novelty induced hyperactivity, spatial novelty preference (SNP) and spatial working memory (SWM). Electrophysiology recordings were made from all animals during novelty-induced hyperactivity and from the second cohort during SNP and SWM testing. For the experiments female and male mice were used. Our previous studies revealed no sex differences in the behavioral performance of WT and *Gria1*^–/–^ mice in the T-Maze and spatial novelty preference test used in this study, or on the pronounced novelty induced hyperactivity of *Gria1*^–/–^ animals ^10^. For the hand-run maze-tasks (SNP, SWM) the experimenter was blind to group-identity.

***Spatial working memory (SWM)***

*Food restriction*

To motivate animals to consume food rewards, access to food was restricted to limit weights to 85% of the free-feeding weight. Animals had *ad libitum* access to water at all times.

*Habituation to equipment*

Animals were habituated to the wooden T-maze (start arm: width 10 cm, length 47 cm; goal arms: width 10 cm, length 35 cm; wall-height 10 cm; elevated 100 cm above ground) for several days, and familiarized with the condensed milk food reward (mixed 50:50 with drinking water). During these habituation sessions, animals were initially allowed to explore the entire maze, consuming food rewards at the ends of each of the arms. Then animals were habituated to the sliding doors by closing one of the goal arm doors (in a pseudo-random order) to force the animal into a goal arm to obtain a condensed milk food reward. Animals were trained to return to the “start box” to collect another condensed milk reward where they would be transiently enclosed. Testing commenced once all animals were running (and eating) without hesitation in the goal arms and returning to the “start box”. During habituation sessions animals were not tethered.

*Rewarded alternation testing*

Animals were given 10 trials of rewarded alternation each day for 7 consecutive days. Animals were tethered to enable simultaneous electrophysiology recordings. Animals were placed onto the central arm (goal arms both closed) and left to freely enter the “start box”. The first trial would start once the animal had consumed the initial reward in the start box. Each trial consisted of a sample and a choice run. In the sample run, one of the goal arms was blocked, forcing the animal into the left or right goal arm. After the mice had consumed the reward in the goal arm they returned to the “start box” to consume another reward. Animals were enclosed in the “start box” for an intra-trial interval (delay) of 5 – 10 sec before the choice run was initiated. In the choice run, both of the goal arms were accessible and animals were rewarded for choosing the goal arm that they did not previously visit in the sample run (i.e. for alternating). Then animals returned to the 'start box' to receive a reward and waited for 30 – 45 sec (inter-trial interval) before initiation of the next trial. On each trial, the identity of the sample (i.e. left or right) was determined by a pseudo-random sequence with equal numbers for left and right and no more than 3 trials in a row of the same direction.

During testing, the location of the animal was tracked with ANY-maze (Stoelting). A “decision zone” was defined within the start arm (see Figure 2a). Entry into and exit from the decision zone was detected by ANY-maze, and the ANY-maze interface (AMi) was used to generate 6 distinct TTL pulses that indicated the timing of each trial and the position of the mouse in subzones of the T-maze. Those TTL pulses were fed via an HDMI-coupled I/O-board into the analog inputs of an Open EPhys (www.open-ephys.org, MA, US) acquisition box to time-stamp the animals’ location in the maze at a given time onto the electrophysiological recording file (see below).

***Novelty-induced hyperactivity***

Animals were tethered to enable electrophysiology recordings and then placed into a novel environment consisting of a clear plastic cage containing clean sawdust (length 43 cm, width 22 cm, height 20 cm). Animals were allowed to explore for 5 min. The animals’ location in the open field was tracked with ANY-maze (Stoelting, UK) and the distance travelled was calculated in 1 min time bins.

***Spatial novelty preference in the Y-maze***

In an unfamiliar room, animals were tethered and placed into the start arm of a clear Perspex Y-maze (each arm: width 8 cm, length 30 cm, height 20 cm) containing a mixture of clean and dirty sawdust (from unfamiliar mice of the same sex) at a ratio of 2:1. Testing consisted of a sample and a test phase. During the sample phase, mice were allowed to explore the start arm and one of the goal arms of the Y-maze (arm allocation counterbalanced for group) for 5 min. Mice were then removed from the maze for an intra-trial interval (ITI) of 1 min. During the ITI, the sawdust within the Y-maze was redistributed (to mix up any odor trails). During the test phase, animals were returned to the start arm of the Y-maze and allowed to explore all arms of the Y-maze for 5 min. The animals’ location was tracked with ANY-maze and the time spent within and the number of entries into the arms were calculated. The test phase was conducted for 5 min to collect electrophysiological data of the same length as for the sample phase, but analysis of the behavioral preferences was made from the first minute only to avoid confounds from habituation to the novel goal arm. The position of the animal in different zones of the maze was coded into different TTL-outputs from the ANY-maze interface (AMi, Stoelting, UK) and recorded by the Open-EPhys acquisition board simultaneously to the LFP-recordings (as described above for the T-maze).

**Electrophysiology and analysis**

***Data acquisition and pre-processing***

Data were amplified and digitized with an RHD2132 headstage (Intan Technologies, CA, US) connected to an Open-Ephys acquisition board using a sampling rate of 15 kHz and a bandpass-filtering between 0.1 and 300 Hz. A custom-made adaptor interfacing between the implanted connector and the headstage was wired so that all acquired channels were referenced to ground (screw over the contralateral cerebellum), while an additional reference signal (surface EEG over contra-lateral motor cortex) was recorded independently, but not used for the present analysis. Data were exported to Matlab (MathWorks), down-sampled to 1 kHz and analyzed with custom-written scripts. To reduce low frequency drift, signals were first detrended using the *locdetrend* function of the Chronux Toolbox (http://chronux.org/) with 1 s of data and a sliding window of 0.5 s, and then bandpass-filtered between 1-250 Hz. Animals were excluded from the electrophysiological analysis, if their signal was obviously corrupted (e.g. due to connection between multiple channels, loss of insulation, or high noise levels).

***Coherence and power of LFPs during spatial working memory***

Timestamps for decision zone entry and exit were calculated based on the TTL outputs from ANY-maze. The median time spent in the decision zone during sample and choice runs was calculated (across all training blocks) and found to be between 1.2–2 s. For each trial a 1.2 s segment of LFP data from the dorsal hippocampus (dHipp) and prefrontal cortex (PFC) was taken immediately prior to the “decision zone” exit of the sample and choice run of each trial. Trials were excluded if the animal made repeated entries into the decision zone during the sample or choice run. In addition, all trials where the time in the decision zone was < 0.25 s were excluded as these were indicative of rare instances of mis-tracking by ANY-maze. Finally, the standard deviation of the LFP signal was calculated for all trials. Sample or choice trials were excluded if the LFP signal drifted above or below 3.5 standard deviations of the mean LFP fluctuation, indicating a large drift in the signal, or a noise spike.

***Coherence analysis***

Coherence was calculated using the *coherencyc* function in the Chronux toolbox (http://chronux.org/). Coherence between the PFC and dHipp was calculated for sample and choice trials between 0-­48 Hz. Within animals, the inbuilt trial averaging function was used to create an average coherence spectrum for all trials over the 7 testing blocks.

A clear peak in the theta frequency (often considered to be between 4-12 Hz in rodents) was observed around 8-9 Hz, and a range of 6-12 Hz was used as theta range for all analysesyz. Given that this peak, reflecting the dominant and frequency-stable theta coherence in the network, was so clearly identifiable we determined *peak theta coherence*, i.e. the amplitude of the peak in the theta-range of the coherogram as the primary measure of theta coherence as done in other studies ^11–13^. We also determined the average frequency at which this peak occurred as *peak theta coherence frequency*. Additionally, we also used *mean theta coherence* as the average coherence amplitude in the 6-12 Hz bracket, as done in a different set of studies ^14–16^. Delta, beta and low-gamma frequency coherence were defined as the *mean* coherence in the 1-4, 20-30 and 30-48 Hz range respectively, given that a characteristic peak was often lacking in these frequency ranges. Group average coherence plots were generated by averaging the coherence spectra generated for each individual animal within each group.

***Power calculations during SWM***

Power was assessed via the multi-taper approach using the *mtspectrumc* function (http://chronux.org/). LFP power within the PFC and dHipp was calculated for sample and choice trials between 0-48 Hz. As for coherence (see above), *peak* and *mean* theta power was calculated, given the presence of a clearly identifiable peak in the theta range (6-12 Hz) of the power spectra.

***Coherence and power of LFPs during testing of locomotor activity***

To visualize changes in the power of local oscillations and the coherence between mPFC and dHipp, spectrograms and coherograms were generated using the *mtspecgramc* and *cohgramc* functions, respectively (http://chronux.org/), during 5 min of exploration in a novel environment. Power values were expressed as 10 x log_10_ values for all analyses. For group comparisons, the overall power and coherence was calculated between 0-48 Hz over the course of the entire 5 min exploration period using the *mtspectrumc* and *coherencyc* functions (http://chronux.org/). For calculations, the power and coherence spectra were smoothed (50 points) and the mean delta (1-4 Hz), beta (20-30 Hz) and gamma (30-48 Hz) values were calculated. In the theta range, there was a reliable and prominent peak, so the amplitude of that peak (maximum value within 6-12 Hz) was measured to assess theta power, rather than the mean value across the interval. Consistent with some previous studied we also extracted the mean theta (6-12 Hz) power and coherence (see above).

***Variability of dHipp theta power and frequency***

To calculate the variations in peak theta power frequency in the dorsal hippocampus, changes in power over time were calculated between 6-12 Hz using 2 s of data with a 2 s sliding window (i.e.non-overlapping). The peak theta amplitude and its associated frequency was extracted across time and used to calculate the coefficient of variance (CV = 100 * standard deviation/mean) for each animal.

***Theta-gamma cross-frequency coupling***

Phase-amplitude coupling of low-gamma (30-48 Hz) to theta (6-12 Hz) oscillations was calculated using the *PAC* matlab script previously published by Dr. Bradley Voytek ^17^. Prior to phase amplitude coupling analysis the signals were detrended (as described above) and z-scored.

***Coherence and power of LFPs during spatial novelty preference***

Coherence and power were calculated as above. To enable a within-task comparison, coherence and power were calculated during entries to either the novel or the familiar arm (with the minimum entry time set to 2 s) during the 5-min test phase. We analyzed the full 5 min of data (compared to the first 1 min for behavioral assessment) to have enough novel and familiar arm entries to reliably calculate coherence and power.

**Statistical analysis**

Data were analyzed in SPSS (IBM, NY, US) and GraphPad Prism (GraphPad Software, CA, US). Unless stated otherwise, differences between the three experimental subgroups *Gria1*^–/–^, viral hippocampal rescue of GLUA1 expression (GLUA1^CA2/3^), and littermate wild-type control (WT) mice were assessed with univariate or, where applicable, repeated-measures ANOVA. All subsequent post-hoc tests were Tukey-HSD. Data from male and female mice were combined for analysis. See Supplementary Table S1 for full details of statistical tests.

# SUPPLEMENTARY TABLE: Statistics

Abbreviations: RM, repeated measures ANOVA; UNIV, univariate ANOVA; n = number of animals in the stated sub-group; df1, df2, degrees of freedom in the ANOVA; P, p-value of the ANOVA for the given comparison indicating the level of significance; F-value of the ANOVA for the given comparison

| **Test variable** | **Test type** | **n** | | | **Group** | | | **Training block** | | | **Group * Training block interaction** | | |
| --- | --- | --- | --- | --- | --- | --- | --- | --- | --- | --- | --- | --- | --- |
|  |  | **WT** | ***Gria1*^-/-^** | **GLUA1^CA2/3^** | ***df1, df2*** | ***F*** | ***P*** | ***df1, df2*** | ***F*** | ***P*** | ***df1, df2*** | ***F*** | ***P*** |
| SWM performance  (blocks 1-7) | RM | 13 | 12 | 15 | 2,37 | 65.430 | <0.001 | 6,222 | 2.886 | 0.010 | 12,222 | 0.840 | 0.609 |
| SWM performance  (blocks 1-3) | RM | 13 | 12 | 15 | 2,37 | 54.451 | <0.001 | 2,74 | 5.712 | 0.005 | 4,74 | 0.908 | 0.464 |
| SWM performance  (blocks 4-7) | RM | 13 | 12 | 15 | 2,37 | 36.669 | <0.001 | 3,111 | 0.772 | 0.512 | 6,111 | 0.198 | 0.977 |
| SWM performance  (mean 4-7) | UNIV | 13 | 12 | 15 | 2,37 | 36.669 | <0.001 | - | - | - | - | - | - |

**Supplementary Table S1, section 1: Spatial working memory (SWM) performance on the T-maze (rewarded alternation)**

| **Test variable** | **Test type** | **n** | | | **Group** | | | | **Task phase**  **(sample or choice)** | | | | **Group * Task phase interaction** | | | |
| --- | --- | --- | --- | --- | --- | --- | --- | --- | --- | --- | --- | --- | --- | --- | --- | --- |
|  |  | **WT** | ***Gria1*^-/-^** | **GLUA1^CA2/3^** | ***df1, df2*** | ***F*** | ***P*** | ***df1, df2*** | | ***F*** | ***P*** | ***df1, df2*** | | ***F*** | ***P*** |  |
| dHipp-PFC delta coherence (1-4 Hz) | RM | 7 | 6 | 6 | 2,16 | 3.877 | 0.042 | 1,16 | | 2.384 | 0.142 | 2,16 | | 0.798 | 0.467 |  |
| dHipp-PFC peak theta coherence (6-12 Hz) | RM | 7 | 6 | 6 | 2,16 | 0.536 | 0.595 | 1,16 | | 5.528 | 0.032 | 2,16 | | 1.440 | 0.266 |  |
| dHipp-PFC mean theta coherence (6-12 Hz) | RM | 7 | 6 | 6 | 2,16 | 1.421 | 0.27 | 1,16 | | 2.636 | 0.124 | 2,16 | | 0.16 | 0.853 |  |
| dHipp-PFC beta coherence (20-30 Hz) | RM | 7 | 6 | 6 | 2,16 | 17.41 | <0.0005 | 1,16 | | 17.51 | 0.001 | 2,16 | | 1.864 | 0.187 |  |
| dHipp-PFC low-gamma coherence (30-48 Hz) | RM | 7 | 6 | 6 | 2,16 | 30.05 | <0.0005 | 1,16 | | 1.617 | 0.222 | 2,16 | | 0.329 | 0.724 |  |
| dHipp-PFC delta coherence, choice phase | UNIV | 7 | 6 | 6 | 2,16 | 2.127 | 0.152 | - | | - | - | - | | - | - |  |
| dHipp-PFC peak theta coherence, choice phase | UNIV | 7 | 6 | 6 | 2,16 | 0.469 | 0.634 | - | | - | - | - | | - | - |  |
| dHipp-PFC mean theta coherence, choice | UNIV | 7 | 6 | 6 | 2,16 | 1.410 | 0.273 | - | | - | - | - | | - | - |  |
| dHipp-PFC beta coherence, choice | UNIV | 7 | 6 | 6 | 2,16 | 20.55 | <0.0005 | - | | - | - | - | | - | - |  |
| dHipp-PFC low-gamma coherence, choice | UNIV | 7 | 6 | 6 | 2,16 | 18.63 | <0.0005 | - | | - | - | - | | - | - |  |
| PFC delta power (1-4 Hz) | RM | 7 | 6 | 6 | 2,16 | 1.563 | 0.240 | 1,16 | | 34.72 | <0.0005 | 2,16 | | 2.455 | 0.118 |  |
| PFC peak theta power (6-12 Hz) | RM | 7 | 6 | 6 | 2,16 | 0.770 | 0.480 | 1,16 | | 17.59 | 0.001 | 2,16 | | 1.745 | 0.206 |  |
| PFC mean theta power (6-12 Hz) | RM | 7 | 6 | 6 | 2,16 | 1.328 | 0.293 | 1,16 | | 20.22 | <0.0005 | 2,16 | | 1.794 | 0.198 |  |
| PFC beta power (20-30Hz) | RM | 7 | 6 | 6 | 2,16 | 0.360 | 0.703 | 1,16 | | 10.36 | 0.005 | 2,16 | | 0.764 | 0.482 |  |
| PFC low-gamma power (30-48 Hz) | RM | 7 | 6 | 6 | 2,16 | 0.013 | 0.987 | 1,16 | | 0.106 | 0.748 | 2,16 | | 0.230 | 0.797 |  |
| dHipp delta power (1-4 Hz) | RM | 7 | 6 | 6 | 2,16 | 1.744 | 0.206 | 1,16 | | 19.81 | <0.0005 | 2,16 | | 3.036 | 0.076 |  |
| dHipp peak theta power (6-12 Hz) | RM | 7 | 6 | 6 | 2,16 | 0.946 | 0.409 | 1,16 | | 7.690 | 0.014 | 2,16 | | 0.034 | 0.967 |  |
| dHipp mean theta power (6-12 Hz) | RM | 7 | 6 | 6 | 2,16 | 0.503 | 0.614 | 1,16 | | 7.423 | 0.015 | 2,16 | | 2.059 | 0.160 |  |
| dHipp beta power (20-30Hz) | RM | 7 | 6 | 6 | 2,16 | 4.415 | 0.030 | 1,16 | | 0.489 | 0.495 | 2,16 | | 1.064 | 0.368 |  |
| dHipp low-gamma power (30-48 Hz) | RM | 7 | 6 | 6 | 2,16 | 4.009 | 0.039 | 1,16 | | 0.214 | 0.650 | 2,16 | | 0.099 | 0.906 |  |

**Supplementary Table S1, section 2: Electrophysiology during spatial working memory (SWM) testing on the T-maze (rewarded alternation)**

| **Test variable** | **Test type** | **N** | | | **Group** | | | **Time bin** | | | **Group * Time bin interaction** | | |
| --- | --- | --- | --- | --- | --- | --- | --- | --- | --- | --- | --- | --- | --- |
|  |  | **WT** | ***Gria1*^-/-^** | **GLUA1^CA2/3^** | ***df1, df2*** | ***F*** | ***P*** | ***df1, df2*** | ***F*** | ***P*** | ***df1, df2*** | ***F*** | ***P*** |
| Distance travelled | RM | 17 | 15 | 16 | 2,45 | 16.936 | <0.001 | 4,180 | 5.967 | <0.001 | 8,180 | 3.796 | <0.001 |

**Supplementary Table S1, section 3: Novelty-induced hyperlocomotion (LMA)**

| **Test variable** | **Test type** | **n** | | | **Group** | | | **Time** | | | **Group * Time interaction** | | | |
| --- | --- | --- | --- | --- | --- | --- | --- | --- | --- | --- | --- | --- | --- | --- |
|  |  | **WT** | ***Gria1*^-/-^** | **GLUA1^CA2/3^** | ***df1, df2*** | ***F*** | ***P*** | ***df1, df2*** | ***F*** | ***P*** | ***df1, df2*** | ***F*** | ***P*** |  |
| dHipp delta power (1-4 Hz) | UNIV | 15 | 13 | 12 | 2,37 | 1.798 | 0.180 |  |  |  |  |  |  |  |
| dHipp peak theta power (6-12 Hz) | UNIV | 15 | 13 | 12 | 2,37 | 22.464 | <0.001 | - | - | - | - | - | - |  |
| dHipp mean theta power (6-12 Hz) | UNIV | 15 | 13 | 12 | 2,37 | 2.684 | 0.082 |  |  |  |  |  |  |  |
| dHipp theta peak frequency | UNIV | 15 | 13 | 12 | 2,37 | 106.547 | <0.001 | - | - | - | - | - | - |  |
| dHipp beta power (20-30 Hz) | UNIV | 15 | 13 | 12 | 2,37 | 9.850 | <0.001 | - | - | - | - | - | - |  |
| dHipp gamma power (30-48 Hz) | UNIV | 15 | 13 | 12 | 2,37 | 4.231 | 0.022 |  |  |  |  |  |  |  |
| PFC delta power (1-4 Hz) | UNIV | 15 | 13 | 12 | 2,37 | 1.675 | 0.201 |  |  |  |  |  |  |  |
| PFC peak theta power (6-12 Hz) | UNIV | 15 | 13 | 12 | 2,37 | 12.352 | <0.001 | - | - | - | - | - | - |  |
| PFC mean theta power (6-12 Hz) | UNIV | 15 | 13 | 12 | 2,37 | 5.252 | 0.010 |  |  |  |  |  |  |  |
| PFC theta peak frequency | UNIV | 15 | 13 | 12 | 2,37 | 0.954 | 0.394 | - | - | - | - | - | - |  |
| PFC beta power (20-30 Hz) | UNIV | 15 | 13 | 12 | 2,37 | 0.927 | 0.405 | - | - | - | - | - | - |  |
| PFC gamma power (30-48 Hz) | UNIV | 15 | 13 | 12 | 2,37 | 1.958 | 0.156 |  |  |  |  |  |  |  |
| dHipp theta power CV analysis (6-12 Hz) | UNIV | 15 | 13 | 12 | 2,37 | 7.655 | 0.002 | - | - | - | - | - | - |  |
| dHipp theta frequency CV analysis (6-12 Hz) | UNIV | 15 | 13 | 12 | 2,37 | 13.313 | <0.001 | - | - | - | - | - | - |  |
| dHipp theta-gamma phase-amplitude coupling | UNIV | 15 | 13 | 12 | 2,37 | 15.23 | <0.001 |  |  |  |  |  |  |  |
| dHipp-PFC delta coherence (1-4 Hz) | UNIV | 15 | 13 | 12 | 2,37 | 0.456 | 0.637 | - | - | - | - | - | - |  |
| dHipp-PFC peak theta coherence (6-12 Hz) | UNIV | 15 | 13 | 12 | 2,37 | 8.356 | 0.001 | - | - | - | - | - | - |  |
| dHipp-PFC mean theta coherence (6-12 Hz) | UNIV | 15 | 13 | 12 | 2,37 | 1.838 | 0.173 |  |  |  |  |  |  |  |
| dHipp-PFC peak theta-coherence frequency | UNIV | 15 | 13 | 12 | 2,37 | 0.972 | 0.388 | - | - | - | - | - | - |  |
| dHipp-PFC beta coherence (20-30 Hz) | UNIV | 15 | 13 | 12 | 2,37 | 0.081 | 0.922 | - | - | - | - | - | - |  |
| dHipp-PFC gamma coherence (30-48 Hz) | UNIV | 15 | 13 | 12 | 2,37 | 0.838 | 0.441 |  |  |  |  |  |  |  |
| dHipp peak theta power (start vs. end) | RM | 15 | 13 | 12 | 1,37 | 2.896 | 0.068 | 1,37 | 8.69 | 0.006 | 2,37 | 8.408 | 0.001 |  |
| dHipp mean theta power (start vs. end) | RM | 15 | 13 | 12 | 1,37 | 0.518 | 0.600 | 1,37 | 2.679 | 0.110 | 2,37 | 3.907 | 0.029 |  |
| PFC peak theta power (start vs. end) | RM | 15 | 13 | 12 | 1,37 | 0.682 | 0.512 | 1,37 | 3.455 | 0.071 | 2,37 | 0.533 | 0.591 |  |
| PFC mean theta power (start vs. end) | RM | 15 | 13 | 12 | 1,37 | 0.758 | 0.474 | 1,37 | 9.188 | 0.004 | 2,37 | 0.539 | 0.588 |  |
| dHipp peak theta coherence (start vs. end) | RM | 15 | 13 | 12 | 1,37 | 0.407 | 0.660 | 1,37 | 5.084 | 0.030 | 2,37 | 5.447 | 0.008 |  |
| dHipp mean theta coherence (start vs. end) | RM | 15 | 13 | 12 | 1,37 | 0.403 | 0.671 | 1,37 | 0.022 | 0.884 | 2,37 | 3.464 | 0.042 |  |
| dHipp-PFC peak theta coherence; interval analysis | RM | 15 | 13 | 12 | 2,37 | 3.333 | 0.047 | 29,1073 | 1.473 | 0.052 | 58,1073 | 1.542 | 0.007 |  |
| dHipp-PFC mean theta coherence; interval analysis | RM | 15 | 13 | 12 | 2,37 | 1.639 | 0.208 | 29,1073 | 1.463 | 0.055 | 58,1073 | 1.291 | 0.074 |  |
| dHipp-PFC peak theta coherence; interval analysis (WT and GLUA1^CA2/3^ combined) | RM | 15 | - | 12 | - | - | - | 29,754 | 1.998 | 0.002 | - | - | - |  |
| dHipp-PFC peak theta coherence; interval analysis (*Gria1*^-/-^) | RM | - | 13 | - | - | - | - | 29,348 | 1.758 | 0.011 | - | - | - |  |

**Supplementary Table S1, section 4: Electrophysiology during novelty-induced hyperlocomotion in the open field**

| **Test variable** | **Test type** | **n** | | | **Group** | | | **Arm**  **(Novel or Familiar)** | | | **Group * Arm Interaction** | | |
| --- | --- | --- | --- | --- | --- | --- | --- | --- | --- | --- | --- | --- | --- |
|  |  | **WT** | ***Gria1*^-/-^** | **GLUA1^CA2/3^** | ***df1, df2*** | ***F*** | ***P*** | ***df1, df2*** | ***F*** | ***P*** | ***df1, df2*** | ***F*** | ***P*** |
| SNP  time preference ratio | UNIV | 7 | 5 | 5 | 2,20 | 8.403 | 0.002 | - | - | - | - | - | - |

**Supplementary Table S1, section 5: Behavior during spatial novelty preference testing in the Y-maze**

| **Test variable** | **Test type** | **n** | | | **Group** | | | **Arm**  **(Novel or Familiar)** | | | **Group * Arm**  **Interaction** | | |
| --- | --- | --- | --- | --- | --- | --- | --- | --- | --- | --- | --- | --- | --- |
|  |  | **WT** | ***Gria1*^-/-^** | **GLUA1^CA2/3^** | ***df1, df2*** | ***F*** | ***P*** | ***df1, df2*** | ***F*** | ***P*** | ***df1, df2*** | ***F*** | ***P*** |
| dHipp delta power | RM | 7 | 5 | 5 | 2,14 | 0.039 | 0.962 | 1,14 | 0.752 | 0.400 | 2,14 | 0.227 | 0.800 |
| dHipp peak theta power | RM | 7 | 5 | 5 | 2,14 | 1.438 | 0.270 | 1,14 | 5.790 | 0.031 | 2,14 | 0.753 | 0.489 |
| dHipp mean theta power | RM | 7 | 5 | 5 | 2,14 | 0.484 | 0.626 | 1,14 | 1.858 | 0.194 | 2,14 | 0.142 | 0.868 |
| dHipp beta power | RM | 7 | 5 | 5 | 2,14 | 3.551 | 0.057 | 1,14 | 0.005 | 0.946 | 2,14 | 0.792 | 0.472 |
| dHipp gamma power | RM | 7 | 5 | 5 | 2,14 | 2.213 | 0.146 | 1,14 | 0.03 | 0.866 | 2,14 | 1.591 | 0.238 |
| PFC delta power | RM | 7 | 5 | 5 | 2,14 | 0.300 | 0.746 | 1,14 | 0.222 | 0.645 | 2,14 | 0.422 | 0.664 |
| PFC peak theta power | RM | 7 | 5 | 5 | 2,14 | 2.311 | 0.136 | 1,14 | 4.019 | 0.065 | 2,14 | 0.403 | 0.676 |
| PFC mean theta power | RM | 7 | 5 | 5 | 2,14 | 2.339 | 0.133 | 1,14 | 3.382 | 0.087 | 2,14 | 0.59 | 0.568 |
| PFC beta power | RM | 7 | 5 | 5 | 2,14 | 1.229 | 0.322 | 1,14 | 2.02 | 0.177 | 2,14 | 2.663 | 0.105 |
| PFC gamma power | RM | 7 | 5 | 5 | 2,14 | 0.447 | 0.648 | 1,14 | 0.000 | 0.992 | 2,14 | 0.304 | 0.743 |
| dHipp-PFC delta coherence | RM | 7 | 5 | 5 | 2,14 | 1.345 | 0.292 | 1,14 | 0.038 | 0.848 | 2,14 | 1.841 | 0.195 |
| dHipp-PFC peak theta coherence | RM | 7 | 5 | 5 | 2,14 | 20.845 | <0.001 | 1,14 | 7.235 | 0.018 | 2,14 | 0.999 | 0.393 |
| dHipp-PFC mean theta coherence | RM | 7 | 5 | 5 | 2,14 | 23.21 | <0.0005 | 1,14 | 1.336 | 0.267 | 2,14 | 0.65 | 0.537 |
| dHipp-PFC beta coherence | RM | 7 | 5 | 5 | 2,14 | 12.69 | <0.001 | 1,14 | 0.845 | 0.373 | 2,14 | 0.198 | 0.823 |
| dHipp-PFC gamma coherence | RM | 7 | 5 | 5 | 2,14 | 9.22 | 0.003 | 1,14 | 0.636 | 0.439 | 2,14 | 0.564 | 0.581 |

**Supplementary Table S1, section 6: Electrophysiology during spatial novelty preference testing in the Y-maze.** All analysis use the respective electrophysiological parameter from the novel and the familiar arm visits during the choice phase as dependent variables, i.e. arm (novel vs familiar; within-subject comparison) and group (between-subject comparison) are the independent variables of the RM-ANOVA. Frequency bands are as used for the other tests (see sections 2 and 4).

# SUPPLEMENTARY FIGURES


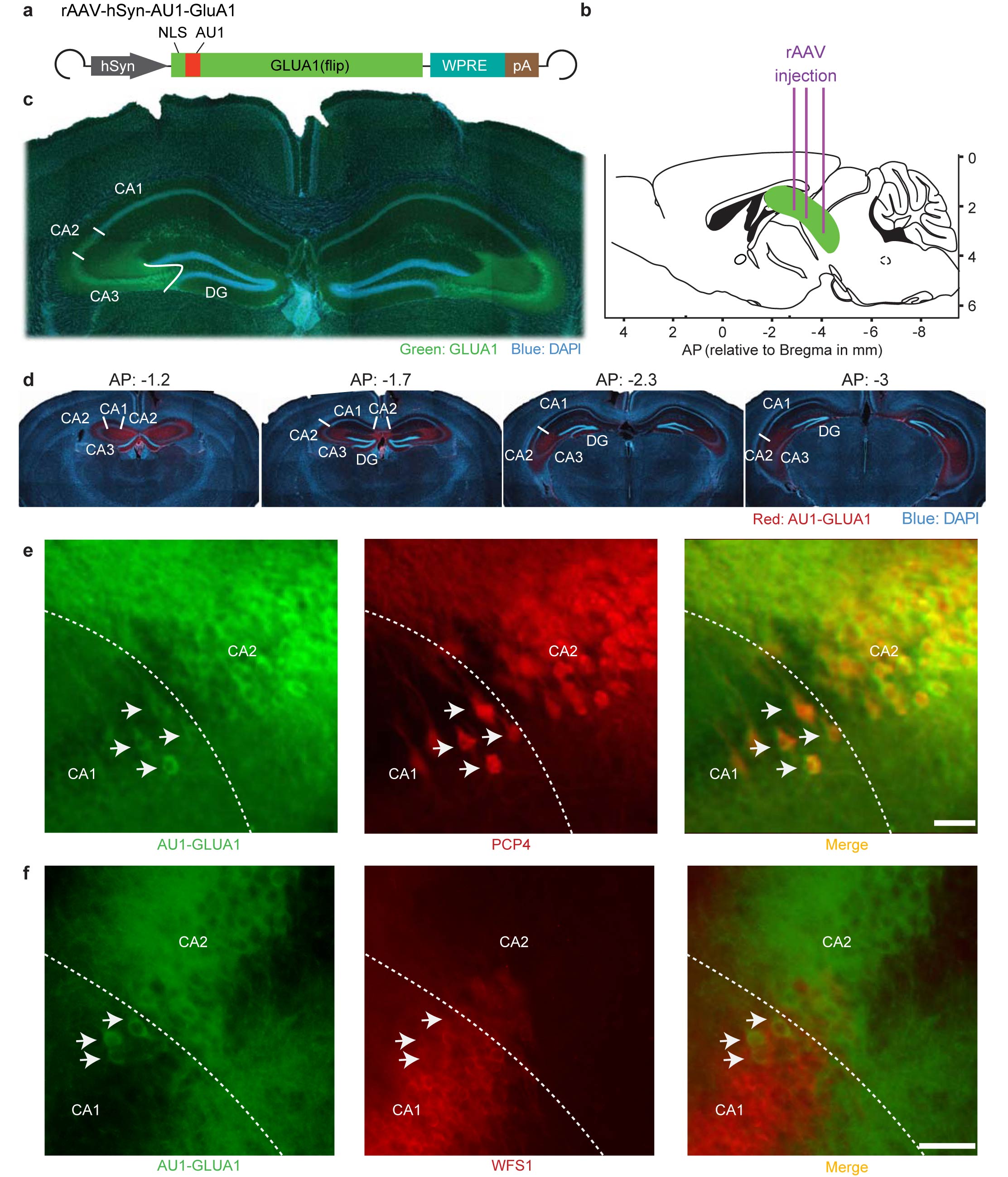


**Supplementary Figure 1.** Viral GluA1 expression is selective to CA2/CA3.

(**a**) Schematic of GLUA1 virus design. (**b**) Injection coordinates with green shaded area indicating the hippocampus. (**c**) Immuno-staining against GLUA1 following injection of the vector in (**a**) into a *Gria1*^-/-^ mouse. Hippocampal subfields CA1-3 and DG are indicated. (**d**) Representative example of AU1-GLUA1 expression (red, staining against AU1) along the anterior/posterior (AP) axis of the hippocampus. Note the absence of expression in CA1 and DG throughout. (**e**) Immuno-staining against AU1 (detecting the AU1-GLUA1 construct; green) and the putative CA2 marker PCP4 (red) indicates their co-localization. The CA1/CA2 border is indicated (white dotted line). Note the PCP4-positive cells scattered in CA1 that are also selectively AU1-GLUA1-positive (white arrows). Scale bar = 50 μm. (**f**) Lack of co-localization of AU1-GLUA1 (green) with the putative CA1 marker WFS1 (red). The CA1/CA2 border is indicated (white dotted line). Note the AU1-GLUA1 positive cells scattered in CA1 that lack expression of WFS1 (white arrows). Scale bar = 50 μm.

#
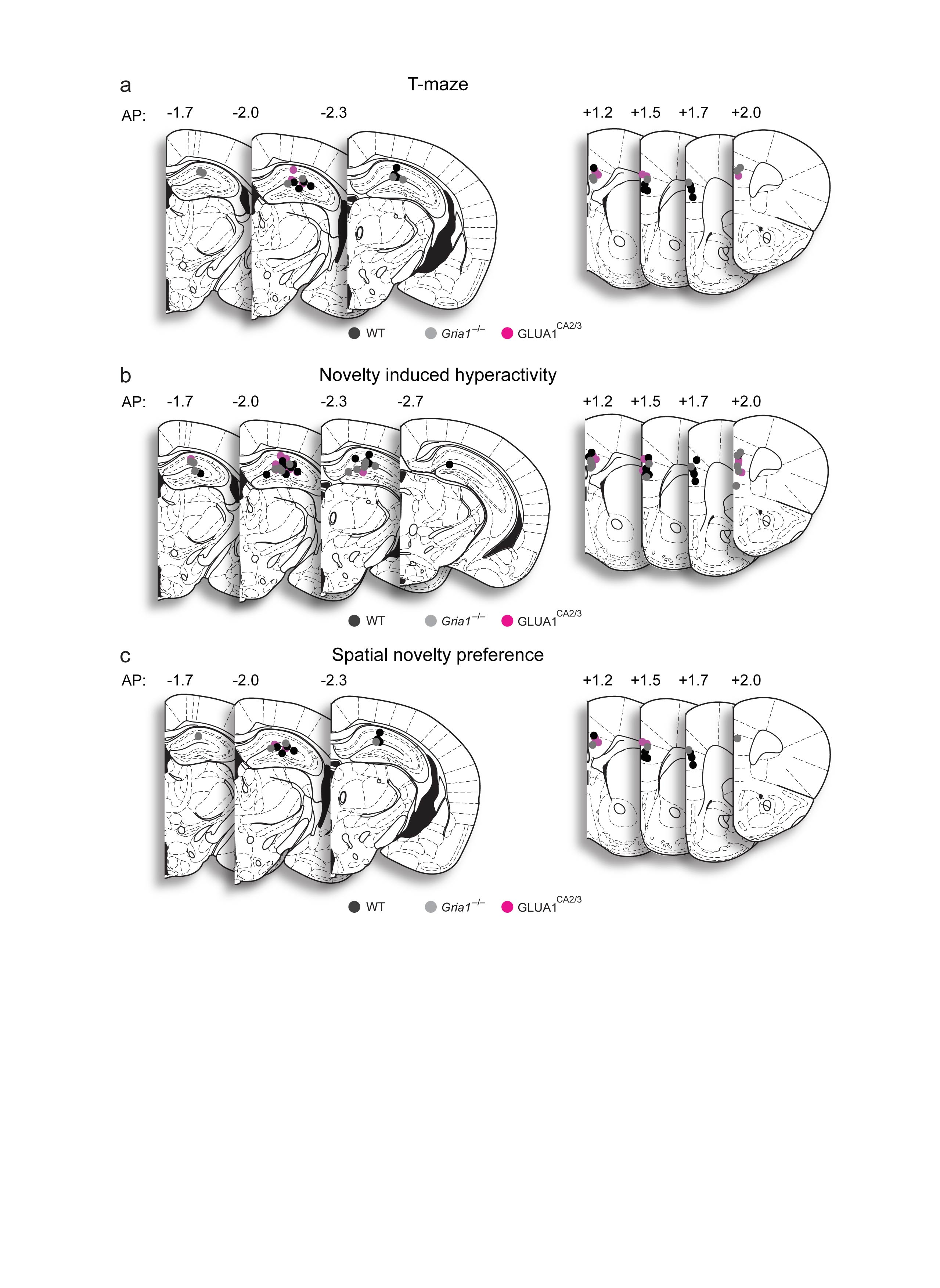


**Supplementary Figure 2.** Electrode placement maps.

Estimates of electrode placements in mice included in T-maze (**a**), Open field (**b**) and Y-maze (**c**) experiments as reconstructed from electrolytic lesions, drawn onto schematics of coronal mouse brain slices according to the mouse brain atlas^18^. We failed to locate the recording sites in the PFC in one GLUA1^CA2/3^ and two WT animals; but as LFP signals looked indistinguishable from recordings of other animals of the same group, electrophysiological data from these three mice were included in our analyses.


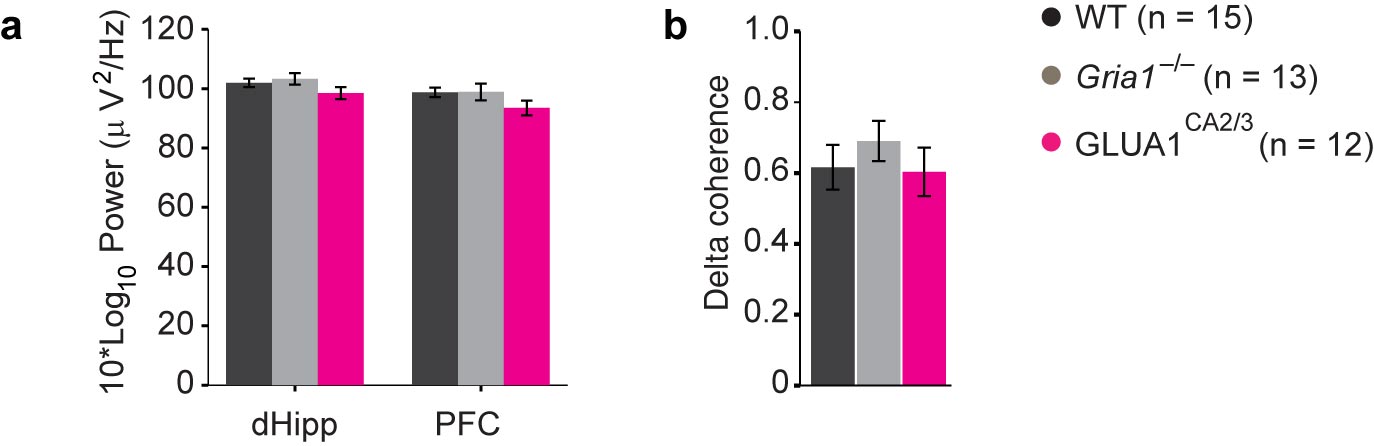


**Supplementary Figure 3.** Delta power and delta coherence in the open field.

(**a-b**) Mean delta (1-4 Hz) power (a) and coherence (b) during the locomotor activity testing in the open field (aggregated over 5 min). Bars display the mean, and error bars the SEM. *N* numbers are indicated in the legend.


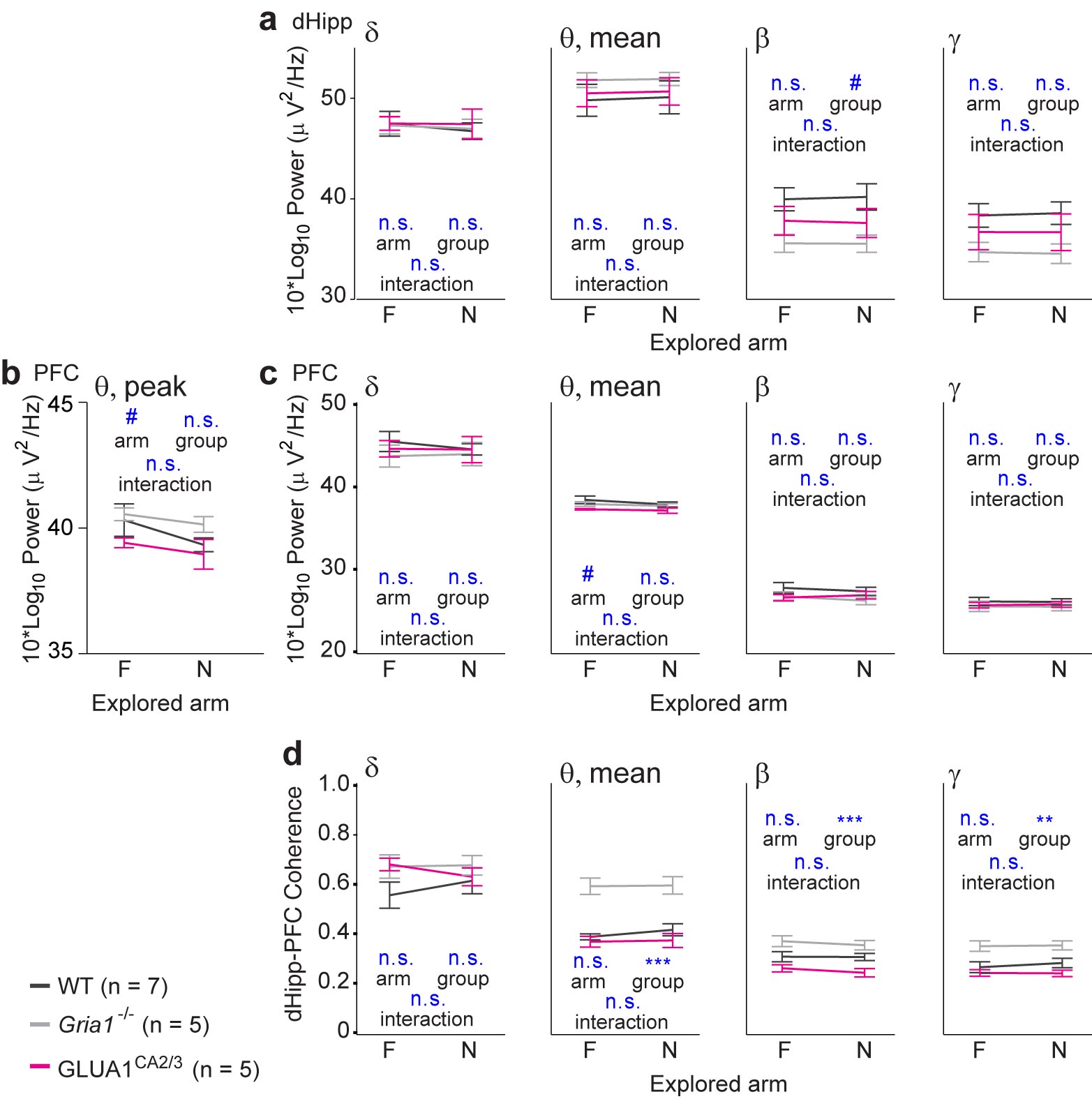


**Supplementary Figure 4.** Local power and coherence during the Y-maze spatial novelty preference task.

(**a, c**) Group averages of *mean* power in the delta (δ), theta (θ), beta (β) and gamma (γ) frequency bands, as indicated, in the dHipp (**a**) or PFC (**c**) during bouts exploring the familiar (F) versus novel (N) Y-maze arm. (**b**) Average peak theta power in PFC during bouts exploring the familiar (F) vs. novel (N) Y-maze arm shown for each animal. (**d**) Group averages of *mean* hippocampal-prefrontal coherence in the delta (δ), theta (θ), beta (β) and gamma (γ) frequency bands, as indicated, during bouts exploring the familiar (F) versus novel (N) Y-maze arm.

Black, wildtype controls (*N* = 7), grey, Gria1^-/-^ (*N* = 5) and magenta, GLUA1^CA2/3^ (*N* = 5); error bars show SEM. Blue symbols show significance level of effects of time, group and group-time interaction (as indicated) obtained in repeated-measures ANOVAs over the 2 time-bins. *** *P* < 0.001, ** *P* < 0.01, # *P* < 0.1, n.s. *P* > 0.1.

# SUPPLEMENTARY DISCUSSION

**Potential confounds by volume conductance and the relation to running speed**

It is possible that coherence measurements are confounded by detection of volume conductance, i.e. changes in amplitudes of extracellular voltage changes get transmitted to another brain region ^19^. To reduce the chance of this impacting our results we placed reference electrodes above the cerebellum, far from the hippocampus, which is the most prominent source of theta oscillations in the brain ^20^. We also have experimental evidence that volume conductance is not driving the effects we see in coherence: Firstly, in all instances where an *increase of coherence* is observed while at the same time *local power* in PFC and dHipp remain the same or even decrease, the coherence measure is clearly not caused by volume conductance. This consideration alone rules out that the task phase-related changes of theta- and beta-coherence we observe on the *T-maze* can be explained by volume conductance. E.g. *mean* theta coherence (Figure 2b) *increases* in the choice phase (relative to sample phase) while mean theta *power* in dHipp (significantly, Figure 1j) and PFC (qualitatively, Figure 1k) even *decreases* in the choice phase. For the *Y-maze*, the large difference in absolute coherence in knockouts relative to the two other groups is not mirrored by local theta oscillations (see Figures 5c and 5d). For the *open-field* experiment there remains the *theoretical* possibility, that the genotype- and time-related differences in theta-coherence could result from the same changes in local theta-oscillations via volume conductance. However, if confound by volume conductance *did exist* in our coherence recordings, it should *always* occur (not just in some experiments), because it is a purely passive mechanism.

There is furthermore discussion on the interrelationship between hippocampal theta (although not theta-coherence) and running speeds ^21^. *Faster* running speed was associated with *enhanced* power and a *higher* peak frequency of hippocampal theta oscillations ^21^. However, Bender et al., Nat Commun, 2015 ^21^, demonstrated that the direction of causality is *from* hippocampal theta *to* running speeds, not the reverse using optogenetic theta-frequency modulation of the medial-septal GABAergic projections that target CA3. This and multiple further studies fit to the framework proposed by our data – namely that hippocampal theta oscillations, controlled by CA3 and its medial-septal afferents, determine theta-coherence and thereby control selective attention (salience attribution) and, hence, exploratory drive which may translate into locomotion. We also show that the link is not necessarily direct (as expected, if attention is the mediator): for example during the choice phase on the T-maze, where theta coherence is greater than in the sample phase (see Figure 2a,b), if anything, animals are running *slower* (see Figure 1d,e). Also, during bouts of exploration of the novel versus the familiar arm of the Y-maze we found elevated coherence (see Figure 5e) at times where the running speed of the animals did not change (data not shown). Finally, regarding local hippocampal theta oscillations, it is interesting to note that *Gria1*^–/–^ animals, which showed the highest locomotor speed (Figure 3b), had even a significantly *lower* peak frequency of hippocampal theta oscillations (Figure 3f), in contrast to what would be expected from a direct link between running speed and hippocampal theta ^21^.

**SUPPLEMENTARY REFERENCES**

1 Zamanillo D, Sprengel R, Hvalby Ø, Jensen V, Burnashev N, Rozov A *et al.* Importance of AMPA Receptors for Hippocampal Synaptic Plasticity But Not for Spatial Learning. *Science* 1999; **284**: 1805–1811.

2 Freudenberg F, Resnik E, Kolleker A, Celikel T, Sprengel R, Seeburg PH. Hippocampal GluA1 expression in Gria1−/− mice only partially restores spatial memory performance deficits. *Neurobiol Learn Mem* 2016; **135**: 83–90.

3 Bannerman DM, Deacon RMJ, Brady S, Bruce A, Sprengel R, Seeburg PH *et al.* A comparison of GluR-A-deficient and wild-type mice on a test battery assessing sensorimotor, affective, and cognitive behaviors. *Behav Neurosci* 2004; **118**: 643–647.

4 Boerner T, Bygrave AM, Chen J, Fernando A, Jackson S, Barkus C *et al.* The group II metabotropic glutamate receptor agonist LY354740 and the D2 receptor antagonist haloperidol reduce locomotor hyperactivity but fail to rescue spatial working memory in GluA1 knockout mice. *Eur J Neurosci* 2017; **45**: 912–921.

5 Sanderson DJ, Hindley E, Smeaton E, Denny N, Taylor A, Barkus C *et al.* Deletion of the GluA1 AMPA receptor subunit impairs recency-dependent object recognition memory. *Learn Mem* 2011; **18**: 181–190.

6 Schmitt WB, Sprengel R, Mack V, Draft RW, Seeburg PH, Deacon RMJ *et al.* Restoration of spatial working memory by genetic rescue of GluR-A–deficient mice. *Nat Neurosci* 2005; **8**: 270–272.

7 Sanderson DJ, Good MA, Skelton K, Sprengel R, Seeburg PH, Rawlins JNP *et al.* Enhanced long-term and impaired short-term spatial memory in GluA1 AMPA receptor subunit knockout mice: Evidence for a dual-process memory model. *Learn Mem* 2009; **16**: 379–386.

8 Shevtsova Z, Malik JMI, Michel U, Schöll U, Bähr M, Kügler S. Evaluation of epitope tags for protein detection after in vivo CNS gene transfer. *Eur J Neurosci* 2006; **23**: 1961–1969.

9 Bygrave AM, Masiulis S, Nicholson E, Berkemann M, Sprengel R, Harrison P *et al.* Knockout of NMDA-receptors from parvalbumin interneurons sensitizes to schizophrenia-related deficits induced by MK-801. *Transl Psychiatry* 2016; **6**: e778.

10 Bannerman DM, Deacon RMJ, Brady S, Bruce A, Sprengel R, Seeburg PH *et al.* A comparison of GluR-A-deficient and wild-type mice on a test battery assessing sensorimotor, affective, and cognitive behaviors. *Behav Neurosci* 2004; **118**: 643–647.

11 Dickerson DD, Wolff AR, Bilkey DK. Abnormal long-range neural synchrony in a maternal immune activation animal model of schizophrenia. *J Neurosci* 2010; **30**: 12424–31.

12 Dickerson DD, Overeem KA, Wolff AR, Williams JM, Abraham WC, Bilkey DK. Association of aberrant neural synchrony and altered GAD67 expression following exposure to maternal immune activation, a risk factor for schizophrenia. *Transl Psychiatry* 2014; **4**: e418.

13 Davis P, Zaki Y, Maguire J, Reijmers LG. Cellular and oscillatory substrates of fear extinction learning. *Nat Neurosci* 2017; **20**: 1624.

14 Adhikari A, Topiwala MA, Gordon JA. Synchronized Activity between the Ventral Hippocampus and the Medial Prefrontal Cortex during Anxiety. *Neuron* 2010; **65**: 257–269.

15 Sigurdsson T, Stark KL, Karayiorgou M, Gogos JA, Gordon JA. Impaired hippocampal-prefrontal synchrony in a genetic mouse model of schizophrenia. *Nature* 2010; **464**: 763–7.

16 O’Neill P-K, Gordon JA, Sigurdsson T. Theta Oscillations in the Medial Prefrontal Cortex Are Modulated by Spatial Working Memory and Synchronize with the Hippocampus through Its Ventral Subregion. *J Neurosci* 2013; **33**: 14211–14224.

17 Voytek B, Canolty RT, Shestyuk A, Crone N, Parvizi J, Knight RT. Shifts in Gamma Phase–Amplitude Coupling Frequency from Theta to Alpha Over Posterior Cortex During Visual Tasks. *Front Hum Neurosci* 2010; **4**. doi:10.3389/fnhum.2010.00191.

18 Franklin K, Paxinos G. *The mouse brain in stereotaxic coordinates*. 3rd ed. Academic Press, 2007.

19 Sirota A, Montgomery S, Fujisawa S, Isomura Y, Zugaro M, Buzsáki G. Entrainment of neocortical neurons and gamma oscillations by the hippocampal theta rhythm. *Neuron* 2008; **60**: 683–97.

20 Buzsáki G, Anastassiou C a., Koch C. The origin of extracellular fields and currents — EEG, ECoG, LFP and spikes. *Nat Rev Neurosci* 2012; **13**: 407–420.

21 Bender F, Gorbati M, Cadavieco MC, Denisova N, Gao X, Holman C *et al.* Theta oscillations regulate the speed of locomotion via a hippocampus to lateral septum pathway. *Nat Commun* 2015; **6**: 8521.
